# Tailoring the Acidity of Liquid Media with Ionizing Radiation: Rethinking the Acid–Base Correlation beyond pH

**DOI:** 10.1021/acs.jpclett.3c00593

**Published:** 2023-05-11

**Authors:** Birk Fritsch, Andreas Körner, Thaïs Couasnon, Roberts Blukis, Mehran Taherkhani, Liane G. Benning, Michael P. M. Jank, Erdmann Spiecker, Andreas Hutzler

**Affiliations:** † Helmholtz Institute Erlangen-Nürnberg for Renewable Energy (IEK-11), Forschungszentrum Jülich GmbH, Cauerstraße 1, 91058 Erlangen, Germany; ‡ Department of Electrical, Electronic and Communication Engineering, Electron Devices (LEB), Friedrich-Alexander-Universität Erlangen-Nürnberg, Cauerstraße 6, 91058 Erlangen, Germany; § Department of Materials Science and Engineering, Institute of Micro- and Nanostructure Research (IMN) and Center for Nanoanalysis and Electron Microscopy (CENEM), Friedrich-Alexander-Universität Erlangen-Nürnberg, Cauerstraße 3, 91058 Erlangen, Germany; ∥ Helmholtz-Zentrum Potsdam, Deutsches GeoForschungsZentrum (GFZ), Telegrafenberg, 14473 Potsdam, Germany; ⊥ Department of Earth Sciences, Free University of Berlin, 12249 Berlin, Germany; # Fraunhofer Institute for Integrated Systems and Device Technology IISB, Schottkystraße 10, 91058 Erlangen, Germany

## Abstract

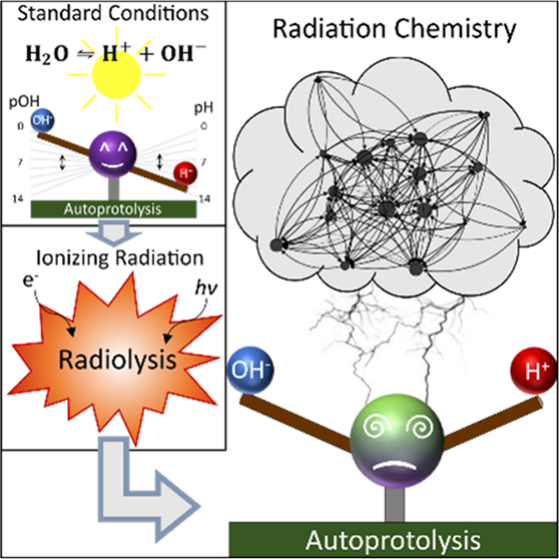

Advanced *in situ* techniques based on electrons and X-rays are increasingly used to gain insights into fundamental processes in liquids. However, probing liquid samples with ionizing radiation changes the solution chemistry under observation. In this work, we show that a radiation-induced decrease in pH does not necessarily correlate to an increase in acidity of aqueous solutions. Thus, pH does not capture the acidity under irradiation. Using kinetic modeling of radiation chemistry, we introduce alternative measures of acidity (radiolytic acidity π* and radiolytic ion product *K*_W_*), that account for radiation-induced alterations of both H^+^ and OH^–^ concentration. Moreover, we demonstrate that adding pH-neutral solutes such as LiCl, LiBr, or LiNO_3_ can trigger a significant change in π*. This provides a huge parameter space to tailor the acidity for *in situ* experiments involving ionizing radiation, as present in synchrotron facilities or during liquid-phase electron microscopy.

*In situ* studies employing ionizing radiation enable unique insights into dynamics on the nanoscale in liquid.^1−4^ Yet, performing reliable cutting-edge research demands precise knowledge of the radiation–matter interaction and the related parameters during the experiment.^5−7^ In particular, when studying chemical phenomena in liquid using electrons (e.g., during liquid-phase transmission electron microscopy (LP-TEM)) or X-rays (e.g., in X-ray diffraction (XRD)) it must be ensured that the effect of radiation on the observation is accounted for.^8−15^

One of the main parameters characterizing the physicochemical properties is the acidity of the liquid phase, generally described by the negative decadic logarithm of the concentration *c*(H^+^) of hydrogen ions, known as pH. Simulations show that electron irradiation of pure water cause a dose-rate dependent increase of *c*(H^+^), thus lowering pH.^14,16^

In contrast, precipitation phenomena observed in aqueous solutions^17^ and analyses of growth kinetics^18^ suggest an elevated concentration of *c*(OH^–^) under irradiation. Likewise, LP-TEM studies on *c*(OH^–^)-driven Si etching are reported.^19,20^ This is unexpected at low pH.

Nevertheless, simultaneous electron-beam induced changes of *c*(H^+^) and *c*(OH^–^) were not yet discussed in the literature. Moreover, the interpretation of pH in an irradiated liquid must be evaluated in general.

Highly reactive radiolysis products and their subsequent deactivation reactions enable diverse reaction pathways which drastically depend on the chemical environment.^8,13,14,21^ In this sense, the impact of additives to pure water on the acidity has not been discussed to date.

In this Letter, we reconsider the interpretation of pH in irradiated liquids by modeling electron beam and X-ray-induced radiation chemistry in pure water. We show that current models of irradiation-induced acidification are insufficient and introduce a more conclusive description of the acidity in irradiated solutions. Furthermore, the impact of different supposedly pH-irrelevant ionic species typically present in LP-TEM like chloride,^13,15,22−24^ bromide,^2,25−27^ and nitrate^4,22,25,28−31^ on the acidity are investigated.

During autoprotolysis, water molecules dissociate into protons *c*(H^+^) and hydroxide ions *c*(OH^–^) changing the respective concentrations until an equilibrium is reached.
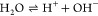
1Considering the law of mass action, an equilibrium constant *K* can be formulated. This can be achieved by regarding the ratio of forward and backward reaction *k*_f_/*k*_b_, as well as via activity α of the respective species:
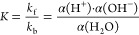
2Due to its normally low dissociation degree, the activity of the solvent (α(H_2_O)) can be assumed to be unity and α(H^+^), α(OH^–^) are approximated by the concentrations *c*(H^+^), *c*(OH^–^).

This leads to the ion product *K*_W_:
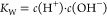
3pH and complementary pOH are defined as the negative decadic logarithms of the H^+^ and OH^–^ concentration normalized to unit molar concentration *c*_unit_ = 1 M:
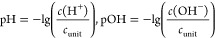
4At standard conditions, the H^+^ concentration of 0.1 μM corresponds to a neutral pH value of 7 in pure water. According to eq 1, also the OH^–^ concentration is 0.1 μM as *c*(H^+^) and *c*(OH^–^) are coupled. Adding acids or bases manipulates *c*(H^+^) and *c*(OH^–^) for the solution to become more acidic or basic, respectively, while maintaining the reciprocal concentration dependency given by eq 3 and illustrated in Figure 1a.

**Figure 1:**
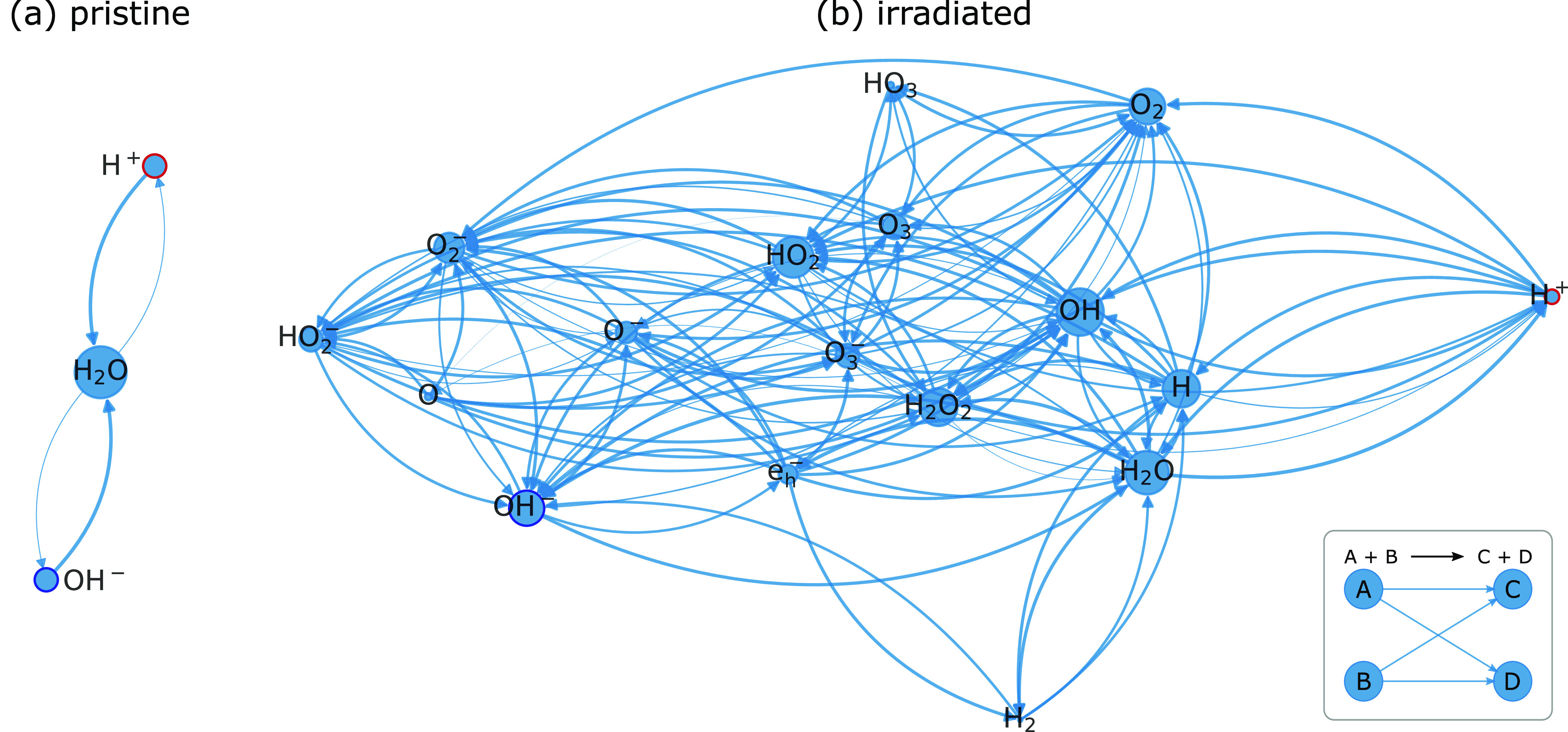
Graph representation of the reaction interplay impacting H^+^ (circled in red) and OH^–^ (circled in dark blue) concentration in (a) pristine and (b) radiolytic water. A tabular representation can be found in Table S2.

In the case of electrons and (hard) X-rays, irradiation of water triggers a relaxation cascade that results in the generation of several primary species within about 1 μs.^32,33^ Those interact with the present environment via subsequent reactions (see the Supporting Information, S1.3, for details). Under such conditions, the inverse proportionality of *c*(H^+^) and *c*(OH^–^) is decoupled by irradiation which contrasts with classical chemical conditions:
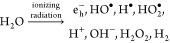
5Consequently, *K*_W_ does not necessarily remain constant in irradiated solutions. Access to the *G*-values of these primary species (amount of substance created per unit energy, see Table S1 of the Supporting Information) is typical for specific types of radiation, and a suitable kinetic model (Table S2 in the Supporting Information) allows one to simulate these reaction pathways.^13,14^ A graph representation^13,34^ can be found in Figure 1b, illustrating the reaction network comprising 17 species coupled over 83 reactions. It becomes directly evident that the amount of possible reaction pathways of H^+^ and OH^–^ are substantially increased compared to the nonirradiated case (Figure 1a).

For pure, aerated (*c*_sat_(O_2_) = 255 μM^14^) water exposed to electron irradiation the steady state concentrations of H^+^ and OH^–^ are plotted as a function of the amount of power absorbed by the specimen (dose rate) (Figure 2a). Evidently, the concentrations of H^+^ and OH^–^ are strongly influenced by reactions with such radiolysis products.

**Figure 2:**
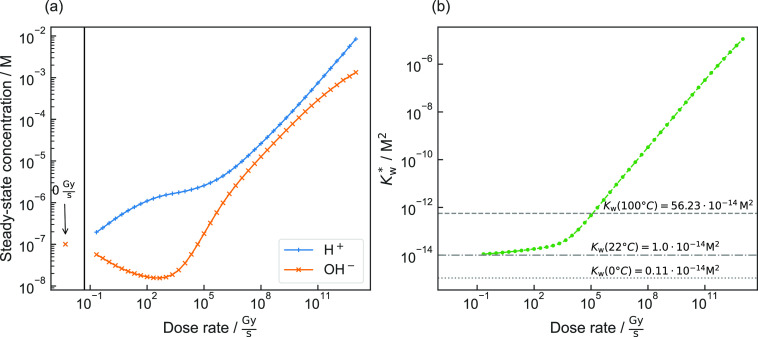
(a) Steady-state concentrations of H^+^ and OH^–^ in pure, aerated water and (b) respective radiolytic ion product, both as a function of the dose rate of electron irradiation compared to changes in *K*_W_ with temperature.

Furthermore, H^+^ and OH^–^ themselves are primarily generated (eq 5; see Supporting Information, section S1.3) so that the ion product is remarkably changed when the solution is exposed to ionizing radiation. As illustrated in Figure 2b, the ion product under irradiation does not denote the equilibrium constant but the product of *c*(H^+^) and *c*(OH^–^) instead. To emphasize this fundamental difference, the radiolytic ion product *K*_W_* is introduced:
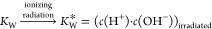
6For dose rates up to about 100 kGy·s^–1^ thermally induced changes in water at ambient pressure could cause similar deviations (Figure 2b).^35^ However, due to the formation of multiple primary species (eq 5), *K*_W_* is the consequence of diverging values for *c*(H^+^) and *c*(OH^–^). This is fundamentally different in the case of thermally excited ion products as here charge and mass balance demand that *c*(H^+^) and *c*(OH^–^) change equally.

A direct proportionality (power law with an exponent of unity) of *K*_W_* to the dose rate is observed for values above 1 kGy·s^–1^ (Figure 2b). The underlying principles of eq 5 and eq 6 are summarized in Figure S1.

Consequently, a more conclusive interpretation of the acidification of irradiated solutions is required that accounts for the drastically different interplay of both species within the solution. To predict whether *c*(H^+^) or *c*(OH^–^) is dominating and, thus, if an irradiated solution constitutes an acidic or basic environment, we introduce the logarithmic ratio of *c*(H^+^) and *c*(OH^–^) as a new measure. This is denoted as radiolytic acidity π*:
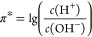
7A π* of zero represents a neutral environment, whereas positive and negative values describe acidic or basic solutions, respectively.

For pure water, *c*(H^+^) and *c*(OH^–^) depend on the initial pH and especially on the type and dose rate of irradiation. For electron exposure the result for a wide range of initial pH and dose rate is visualized in Figure 3. Figure 3a indicates the decoupling of *c*(H^+^) and *c*(OH^–^) under irradiation, while π* is depicted in Figure 3b. Equivalent plots for X-ray exposure are shown in Figure S5 in the Supporting Information. Steady states with a water concentration dropping below 99% of the nonirradiated solution are indicated by unfilled markers, as discussed in the computational methods section.

**Figure 3:**
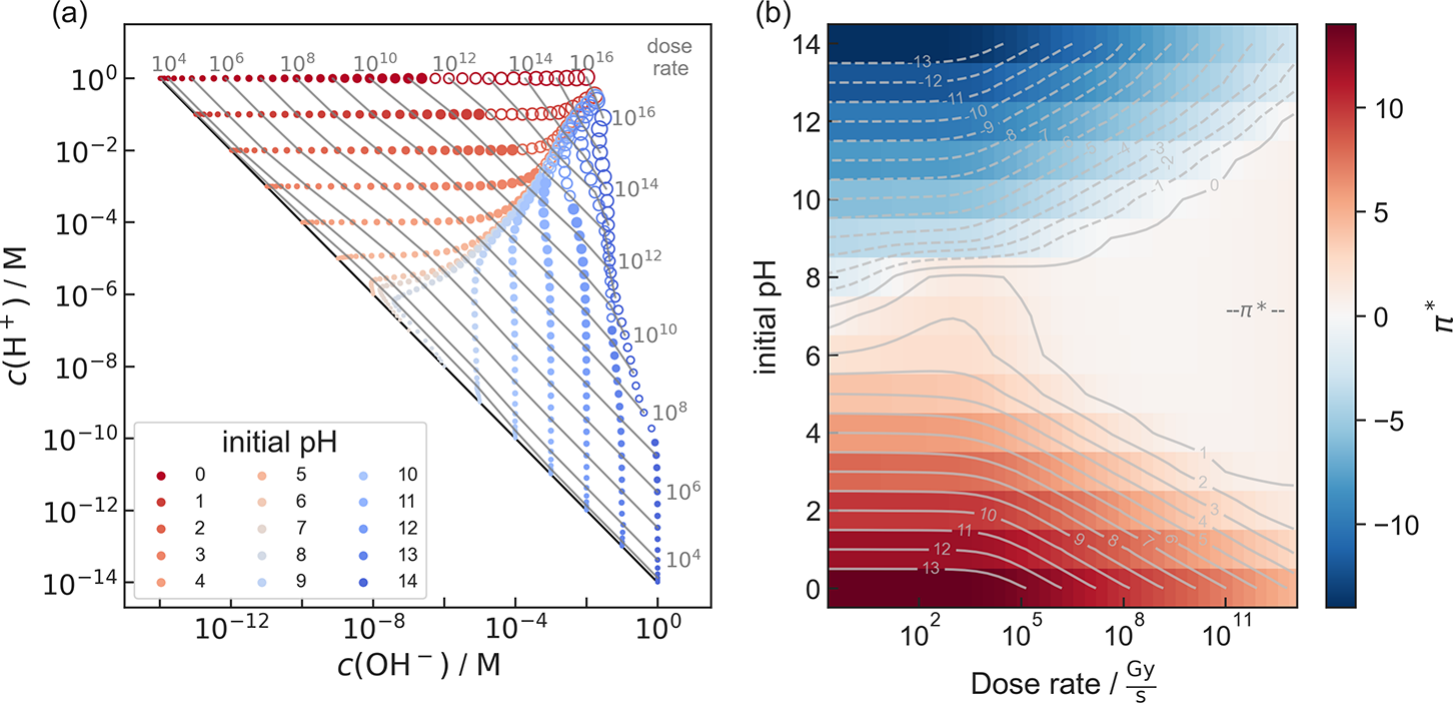
Acid–base chemistry of pure, aerated water as a function of dose rate of an electron beam and the initial pH value. (a) Concentrations of H^+^ and OH^–^ in the steady state. Each dot represents a simulation, while its size is a measure of the dose rate. Dose rate (gray numbers) is given in Gy·s^–1^ and indicated by contour lines. The black diagonal line corresponds to water under equilibrium conditions (*K*_W_ = 10^–14^ M^2^) without irradiation. Empty dots represent simulation results, in which the concentration of water in the steady state drops below 99% of that of nonirradiated solution. (b) π* (color map and gray contour lines) as a function of initial pH and dose rate. A cross-cut at pH 7 can be found in Figure 4. The equivalent plots for X-ray irradiation are shown in Figure S5 in the Supporting Information.

Remarkably, independent of the initial pH value of the specimen solution prior to irradiation, π* converges toward neutral conditions for increasing dose rates. This becomes prominent above ∼1 MGy·s^–1^.

A slight asymmetry favoring acidic conditions is assumed to be related to the (slow) decay of H_2_O_2_ and O_3_ yielding para-oxygen (O, ^3^P). This in turn triggers a reaction cascade in which, beside others, OH^–^ is consumed (see Table S2).

This interplay of radiation chemistry products with acidity highlights the necessity of elucidating the complete reaction chemistry network, which becomes even more pronounced in systems more complex than water. Hence, in the following, the influence of additives on π* is exemplarily demonstrated with LiCl, LiBr, and LiNO_3_.

All simulations are based on radiation chemistry of pure water, with additional reactions and species considered for chlorine, bromine and nitrate-containing solutions (see Supporting Information, section S1.8). As the standard electrode potential of Li^+^ (*E*°(Li/Li^+^) = −3.0401 V)^36^ required for reduction exceed *E*° of the strongest reductant present (*E*°(H_2_O/e_h_^–^) = −2.9 V),^32^ kinetics focusing on the anion–water interplay were considered exclusively.

The evolution of π* for a solution of pH = 7 containing these anions at concentrations of 1 mM and 10 mM is shown in Figure 4. The individual concentrations of *c*(H^+^) and *c*(OH^–^) are separately plotted in the Supporting Information, Figure S7.

**Figure 4:**
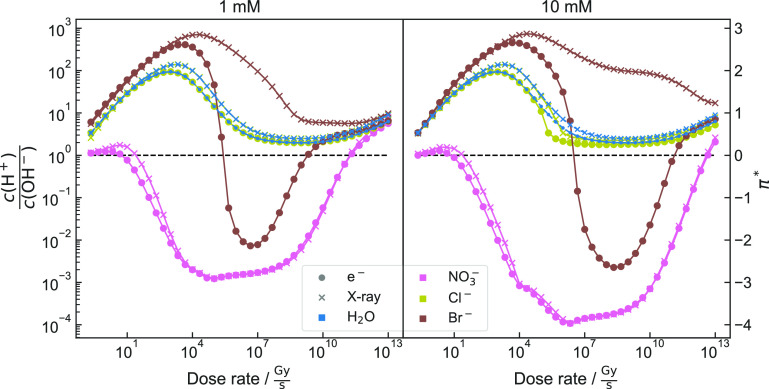
π* of Cl^–^-, Br^–^-, and NO_3_^—^-containing solutions and pure water as a function of dose rate. Anion concentrations of 1 mM (left) and 10 mM (right) are considered. “x”-markers correspond to the simulation performed based on G-values of X-rays. The dotted line corresponds to a balance of *c*(H^+^) = *c*(OH^–^) (π* = 0). Note that for 1 mM, π* for H_2_O and Cl^–^ overlap. Individual simulations and *K*_W_* are denoted in Figures S2 and S7.

As all considered anions represent conjugated bases of strong acids (HCl, HBr, and HNO_3_), their basic strength is generally negligible. Consequently, solutions containing these anions can have a neutral pH prior to irradiation. In combination with thermodynamically stable cations such as Li^+^, the anion-impact on radiation chemistry can be investigated.

Differences in kinetics influence the radiation chemistry, which is evident when comparing dissociation rate constants of conjugated acids (1.46 × 10^10^ s^–1^, HNO_3_ → H^+^ + NO_3_^–^;^37^ 5 × 10^5^ s^–1^, HCl → H^+^ + Cl^–^;^38^ 1 × 10^13^ s^–1^: HBr → H^+^ + Br^–^;^39^ backward reactions are about 3, 6, and 9 orders of magnitude slower).

Nevertheless, simulation results shown in Figure 4 suggest that chloride ions barely influence the evolution of *c*(H^+^) and *c*(OH^–^). However, bromide and nitrate ions strongly alter the acidity of the irradiated solution:

For both radiation types, nitrate mitigates the impact on *c*(H^+^) and *c*(OH^–^) at low dose rates. Above ∼1 MGy·s^–1^, the solution becomes more basic (negative π*) compared to pure water, becomes more neutral for larger dose rates, and finally turns acidic beyond ∼100 GGy·s^–1^_._

While the type of radiation (electrons or X-ray photons) appears to have no qualitative influence on the evolution of *c*(H^+^) and *c*(OH^–^) for aqueous solutions of Cl^–^ or NO_3_^–^, the simulation indicates a difference for Br^–^. When irradiated with X-rays, the solution remains acidic for the entire simulated dose rate range. When considering electron irradiation, however, the solution shows acidic behavior for low and high dose rates, while a basic behavior evolves for dose rates between 500 kGy·s^–1^ and 5 GGy·s^–1^ for an initial concentration of 1 mM.

A change of the concentration from 1 mM to 10 mM does not qualitatively change the shape of the curves but enhances tendencies and therefore shifts intersection points by about 1 to 2 orders of magnitude.

For bromide ion concentrations of 10 mM, this shift causes the intersection point to come close to parameters accessible in standard LP-TEM, indicating that Br^–^ could be a promising candidate for *in situ* studies of acidity-dependent precipitation reactions, using only dose rate adjustments.

The different impact of bromide and chloride is remarkable. Although in general both halides show comparable chemical properties, the larger bromine radical is lower in energy than the chlorine equivalent. This is reflected by the different values of *E*° (+2.43 V for Cl/Cl^–^ and +1.96 V for Br/Br^–^)^40^ which impacts the radical chemistry that dominates kinetic models. Consequently, reaction kinetics of bromide ions exhibit a stronger involvement of acidity-mediating pathways.

By referring to π* in nonirradiated solutions, it can be mapped onto an initial pH value (see Supporting Information Section 1.1 for details). As shown in Figures 2b and S6, *K*_W_* remains close to *K*_W_ for dose rates below 1 kGy. This suggests that approximating the acidity of weakly irradiated solutions by pH may introduce only small errors that could potentially be of minor impact. Nevertheless, pH should not be carelessly translated to irradiated solutions in general, as there, pH does not provide a holistic picture of the acid–base interplay.

Furthermore, the deviations in π* introduced by additives can already become notable at low dose rates (Figure 4). This emphasizes that it is necessary to take the radiolysis-induced reaction networks into account as soon as the solution is exposed to radiation, even if the absolute dose rate is low. Moreover, π* assumes an equivalent reactivity with *c*(H^+^) and *c*(OH^–^). This might be misleading in situations where this prerequisite is not fulfilled. Under such conditions, it is advised to account for *c*(H^+^) and *c*(OH^–^) directly.

In addition, identical π* values can be obtained by different absolute concentrations. Therefore, both, π* and *K*_W_* should be considered in combination as this fully describes the acidity under irradiation.

Note that the analysis presented herein considers steady state concentrations, regardless of the time the system needs to relax into it. Yet, for dose rates relevant to most LP-TEM experiments (above 1 MGy·s^–1^) this occurs within microseconds as demonstrated by the time-dependent analysis of *K*_W_* and π* shown in Figure S3 in the Supporting Information. However, at low dose rates which are accessible using X-rays a notable transient is observed. Nonetheless, substantial changes in π* are notable below one second even for dose rates as low as Gy·s^–1^. This illustrates that radiation effects on the acidity should be considered even at low dose experiments.

Albeit pure, aerated water is the basis of many experiments, this work emphasizes once more that any extrapolation of these findings to different settings must be treated with caution. Although multiple scenarios have already been elucidated here, additional changes in experimental conditions may significantly alter steady state concentrations of H^+^ and OH^–^.

Experimental conditions may deviate significantly from the described simulations. In particular, the simulations shown here consider neither diffusion nor phase boundaries and are therefore only accurate when an isotropic volume element is irradiated homogeneously. Thus, it is only a guidance for experiments using scanning probes in large nonirradiated liquid reservoirs or flow setups. Moreover, radiation-induced formation of additional phases such as gas bubbles or nanocrystals could further alter the steady state concentrations of H^+^ and OH^–^ and hence affect the acidity of the specimen solution.

Mind also that any change of the solvent limits the validity of the assumption that the radiation only interacts with water. This is particularly relevant for high dose rates (usually above ∼10^13^ Gy·s^–1^)^14^ where solvent consumption becomes notable. Likewise, the primary radiation–matter interaction of solutes is not considered here. This approximation appears to be reasonable for small solute concentrations below 1 M only,^33^ as present in this work.

Furthermore, as demonstrated, even additives considered as nonreactive can drastically change the steady-state chemistry at hand. Thus, any extrapolation should be performed cautiously. In this context, it is advised to double-check the assumption of negligible Li^+^ influence on the acidity under irradiation. To do so, we considered a reactivity similarly to the slightly more noble Na^+^ (see Figure S9 and Table S6 in the Supporting Information).^41^ Evidently, the impact on *c*(H^+^) and *c*(OH^–^) remains negligible. As Tesler and Schindelwolf^41^ measured similar rates for Cs^+^, as well, we assume that our findings would also hold for CsCl-, CsBr-, and CsNO_3_-containing solutions.

Especially relevant for LP-TEM is electron beam induced heating, which can significantly affect the redox chemistry. However, this effect is simulated to have a negligible influence on *c*(H^+^) and *c*(OH^–^) in pure water,^42^ suggesting that π* is not affected by beam-heating.

Nevertheless, the herein presented work provides a good approximation for liquid cell architectures with small, static volumes irradiated completely by X-rays (e.g., in synchrotron beamline end stations) and/or electron beams in TEM (e.g., graphene-based liquid cells^43^ and derivatives^25,26,44−48^).

The large parameter space comprising types of radiation, dose rate, additives, initial concentrations etc. allows for tailoring specific conditions. In this Letter, we merely scratch the surface to illustrate the observable effects. However, experimental verification of the model is necessary. Hence, suitable marker reactions, showing structural changes, precipitation, or dissolution in the accessible *c*(H^+^) and *c*(OH^–^) range could be employed.

While our work seconds the finding that irradiation increases *c*(H^+^),^14^ we show that *c*(H^+^) alone is insufficient to quantify the acidity of aqueous solutions interacting with ionizing radiation. Hence, by introducing π* and *K*_W_* as more adequate measures that consider the relation of *c*(H^+^) and *c*(OH^–^), we unveil that, in pure water, electron beam and X-ray irradiation drive the acidity toward a balanced environment, even for high or low initial pH values. Moreover, we show that adding Cl^–^, Br^–^, and NO_3_^–^ ions significantly impacts π*. This allows for tailoring π* during *in situ* experiments by means of initial concentration and dose rate for quantitative studies. In particular, in LP-TEM, Br^–^ ions are promising candidates for validating the predictions made.

Finally, our simulations provide valuable insights for radiation chemistry not only for LP-TEM and X-ray-based techniques but also for any research discipline related to ionizing radiation of aqueous environments. Besides radiolysis, other effects can influence *K*_W_ and consequently the acidity of water, such as temperature (see above) or the Wien effect.^49^ Water electrolysis also exhibits the potential to locally alter the acidity toward a nonequilibrium state, especially dominant in proximity to the electrode surface.^50^ In such cases, regarding the logarithmic ratio *c*(H^+^) and *c*(OH^–^) could be potentially beneficial, as well.

## Computational Methods

Radiolysis simulations were performed utilizing AuRaCh, a custom-build algorithm which has been described in our previous work.^13^ Coupled ordinary differential equations (ODEs) are used to simulate the concentration *c* of species *i* over time *t*, depending on the concentration of reactants *l* and *n*. With the liquid density ρ, dose rate ψ in Gy s^–1^, *G*-value *G*_i_ of *i*, and the rate constant *k*_*j*_ and *k*_*m*_ for reaction *j* and *m*, respectively, the reaction network can be expressed as
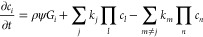
8

Here, we assume sole interaction of radiation with water, for which *G*-values are well-known (Table S1 of the Supporting Information). For electron beam-irradiation the used *G*-values are valid for an energy of 200–300 keV.^14,51,52^ Note that other electron acceleration voltages can have deviating *G*-values which could yield a different outcome.^16,52^ Yet, this can easily be simulated with AuRaCh. The *G*-values for X-ray irradiation are designed for irradiation with high-energy photons^53^ and appear to be reasonable for conventional *in situ* X-ray diffraction.^13^ Simulation results where the amount of radiolytic products in steady state exceeds 1% of the water concentration are labeled appropriately, as here, the assumption of radiation only interacting with the solvent becomes questionable.

Note that the concentration evolution converges against a steady state which is analyzed in this manuscript. An overview about the time required to reach this steady state is provided in Figure S3 in the Supporting Information.

## Data Availability

The reaction sets introduced in this work are available at the AuRaCh GitHub repository: https://github.com/BirkFritsch/Radiolysis-simulations

## Supporting Information Available

Additional considerations, calculations, and kinetic models including reaction sets (PDF)
Transparent Peer Review report available (PDF)
