# Supplementary material for: Tailoring the Acidity of Liquid Media with Ionizing Radiation: Rethinking the Acid–Base Correlation beyond pH
Source: J Phys Chem Lett. 2023 May 11;14(20):4644–51. doi: 10.1021/acs.jpclett.3c00593 (PMC10226122; doi:10.1021/acs.jpclett.3c00593)
Supplement: Supplementary file 1 — jz3c00593_si_001.pdf [file jz3c00593_si_001.pdf]

Supporting Information

Tailoring the Acidity of Liquid Media with Ionizing Radiation: Rethinking the Acid-Base Correlation Beyond pH

Birk Fritsch[1,2,3,*,$], *Andreas Körner*[1,$], *Thaïs Couasnon*[4], *Roberts Blukis*[4], *Mehran Taherkhani*[2], *Liane G. Benning*[4,5], *Michael P. M. Jank*[2,6], *Erdmann Spiecker*[3], *Andreas Hutzler*[1,*]

[1] Forschungszentrum Jülich GmbH, Helmholtz Institute Erlangen-Nürnberg for Renewable Energy (IEK-11), Cauerstraße 1, 91058 Erlangen, Germany
[2] Friedrich-Alexander-Universität Erlangen-Nürnberg, Department of Electrical, Electronic and Communication Engineering, Electron Devices (LEB), Cauerstraße 6, 91058 Erlangen, Germany

[3] Friedrich-Alexander-Universität Erlangen-Nürnberg, Department of Materials Science and Engineering, Institute of Micro- and Nanostructure Research (IMN) and Center for Nanoanalysis and Electron Microscopy (CENEM), Cauerstraße 3, 91058 Erlangen, Germany

[4] Deutsches GeoForschungsZentrum, Helmholtz-Zentrum Potsdam, Deutsches GeoForschungsZentrum (GFZ), Telegrafenberg, 14473 Potsdam, Germany

[5] Department of Earth Sciences, Free University of Berlin, 12249 Berlin, Germany
[6] Fraunhofer Institute for Integrated Systems and Device Technology IISB, Schottkystraße 10, 91058 Erlangen, Germany

[$] These authors contributed equally.
[*]Corresponding author: Birk Fritsch, b.fritsch@fz-juelich.de, Andreas Hutzler, a.hutzler@fz-juelich.de

S1.1 RELATION OF π^* TO pH IN NON-IRRADIATED SOLUTIONS

For non-irradiated solutions, the concentrations of H^+ and OH^- are coupled and their ratio depends on pH. The following equation is designed to be applied in non-irradiated solutions only, therefore it is avoided to call it the decadic logarithm of this ratio π^* here.

$$\begin{aligned}\lg\left(\frac{c(\mathrm{H}^+)}{c(\mathrm{OH}^-)}\right) &= \lg\big(c(\mathrm{H}^+)\big) - \lg\big(c(\mathrm{OH}^-)\big)\\ &= 2\lg\big(c(\mathrm{H}^+)\big) - \lg\big(c(\mathrm{OH}^-)\big) - \lg\big(c(\mathrm{H}^+)\big)\\ &= -2\big[-\lg\big(c(\mathrm{H}^+)\big)\big] - \big[\lg\big(c(\mathrm{OH}^-)\big) + \lg\big(c(\mathrm{H}^+)\big)\big]\\ &= -2\big[-\lg\big(c(\mathrm{H}^+)\big)\big] - \lg\big(c(\mathrm{OH}^-)\cdot c(\mathrm{H}^+)\big) \qquad \text{(S1)}\end{aligned}$$

Inserting equations (3) and (4) into (S1) yields:

$$\lg\left(\frac{c(\mathrm{H}^+)}{c(\mathrm{OH}^-)}\right) = -2\mathrm{pH} - \lg(K_\mathrm{W}) \qquad \text{(S2)}$$

This linear relationship is depicted in Figure S1.

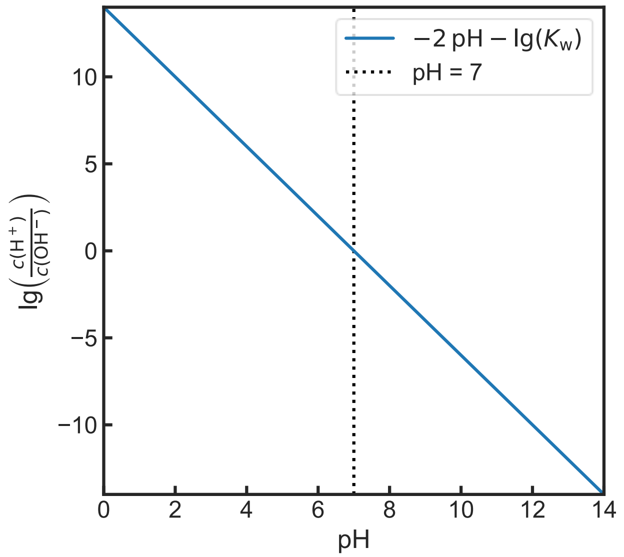

Figure S1:Linear relation of the decadic logarithm of the H^+ and OH^- concentrations at a given pH value for non-irradiated solutions after eq. (S2).

S1.2 RADIOLYTIC ACIDITY π^* AT pH 7.

The simulation for neat, aerated water under standard conditions (no radiation, 25 °C, pH 7) is shown in Figure S2. In this case, π^* remains at positive values between 0.25 and 2 for all dose rates considered. This can be compared to non-irradiated solutions with pH of 6 – 6.875. A π^* of 1 can be considered neutral condition (i.e. the ratio of $c(H^+)$ and $c(OH^-)$ of almost unity). A peak appears 1 $kGy\cdot s^{-1}$ with π^* of about 2, yielding an acidic environment that can be compared to pH 6 in a non-irradiated environment.

The temporal evolution of these steady state concentrations is depicted in Figure S3.

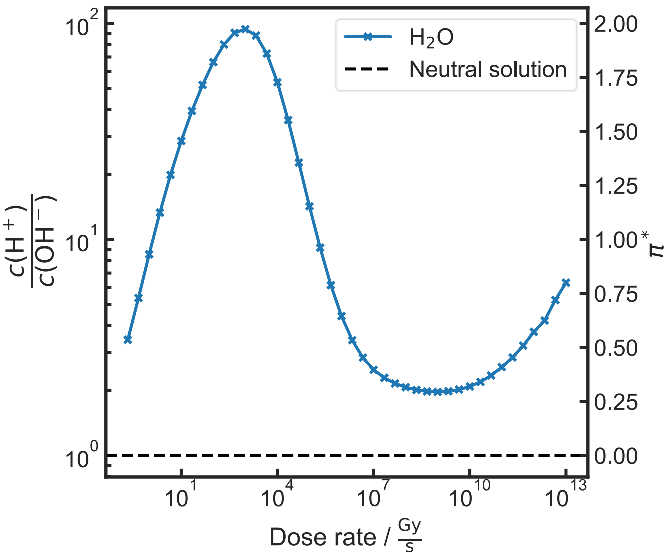

Figure S2:Dependence of the decadic logarithm of the H^+ and OH^- concentrations at pH 7 for irradiated solutions. The data shown here is a zoom of the data presented for pure water in Figure 3.

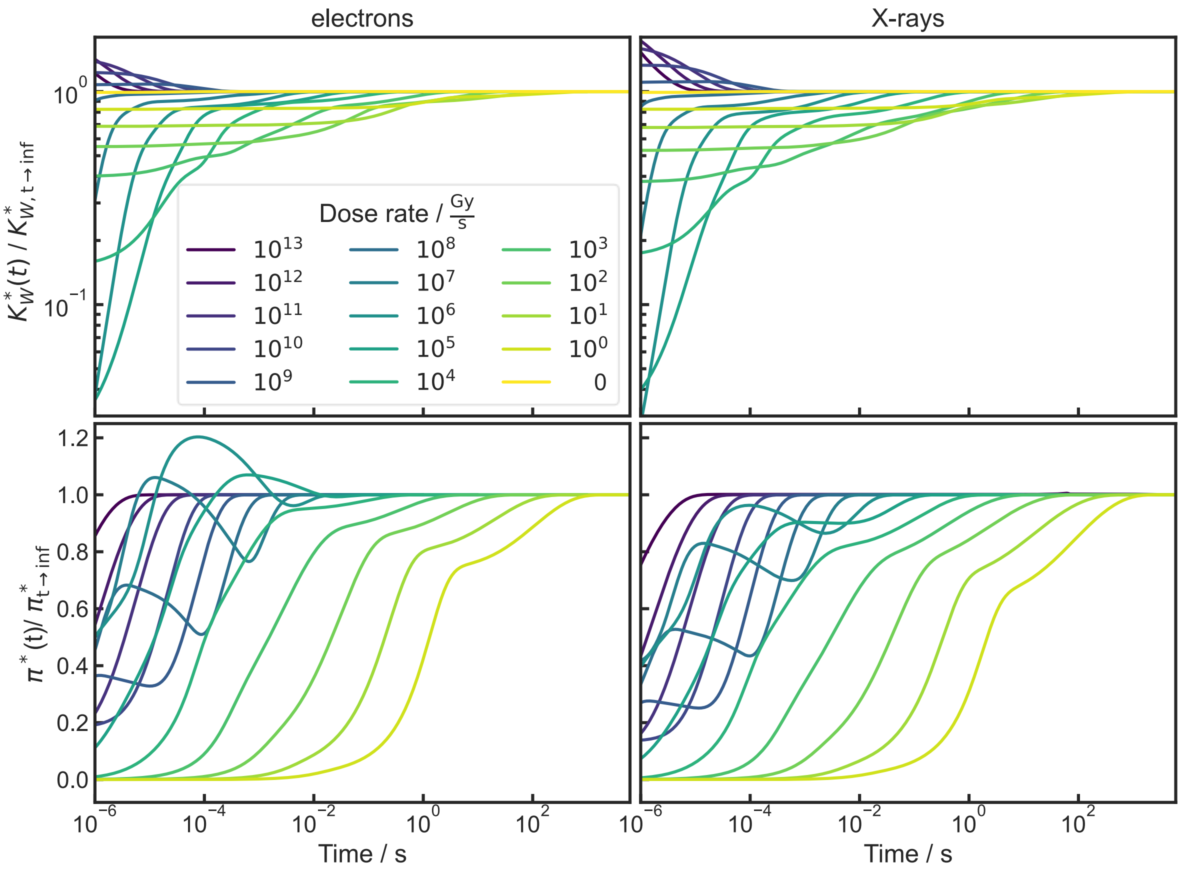

Figure S3: Time dependency of K_W^* (top) and π^* (bottom) for different dose rates (color coded) of electron (left) and X-ray (right) irradiation. Both measures are normalized to the steady state value. The simulation assumes neat, aerated water at an initial pH value of 7. Only values greater than 10^{-6} s are shown because the simulation only provides physically meaningful results after more than 1 µs.

S1.3 INELASTIC RADIATION-MATTER INTERACTIONS

Inelastic interactions between ionizing radiation and water trigger a relaxation cascade that is summarized in Figure S4 which is based on literature.[1,2]. First, the primary energy transfer and rapid electronic relaxation processes occur, causing excitation of high-energy molecular orbitals or molecule ionization in the temporal order of femtoseconds. This so-called 'physical stage' is highly irradiation dependent. The excited products now undergo further relaxation processes including dissociation and first ion-molecule interactions ('physico-chemical stage').

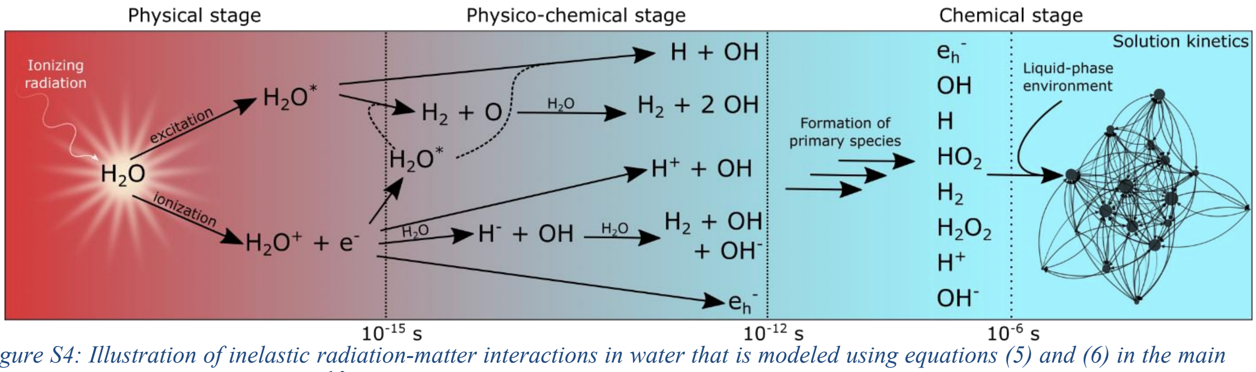

Figure S4: Illustration of inelastic radiation-matter interactions in water that is modeled using equations (5) and (6) in the main manuscript. According to literature.[1,2].

Afterwards, intermolecular interactions dominate the relaxation cascade, as excited energy states are mostly decayed. Hence, this is referred to as 'chemical stage'. A homogeneous distribution of primary species is achieved usually in the µs-range, which are now interacting with the surrounding liquid-phase environment based on the laws of solution kinetics. The ratio of these primary species is described by a set of *G*-values which are determined by the character of the ionizing radiation (Table S1).

Table S1: Generation values used in this work.

	G-value / (Molecules/100 eV)	
primary species	e-beam irrad.[3]	X-ray irrad.[4]
e_h^-	3.47	2.60
H^+	4.42	3.10
OH^-	0.95	0.50
H_2O_2	0.47	0.70
H	1.00	0.66
OH	3.63	2.70
HO_2	0.08	0.02
H_2	0.17	0.45
H_2O	-5.68	-4.64

S1.4 π^* FOR X-RAYS DEPENDENT ON INITIAL pH

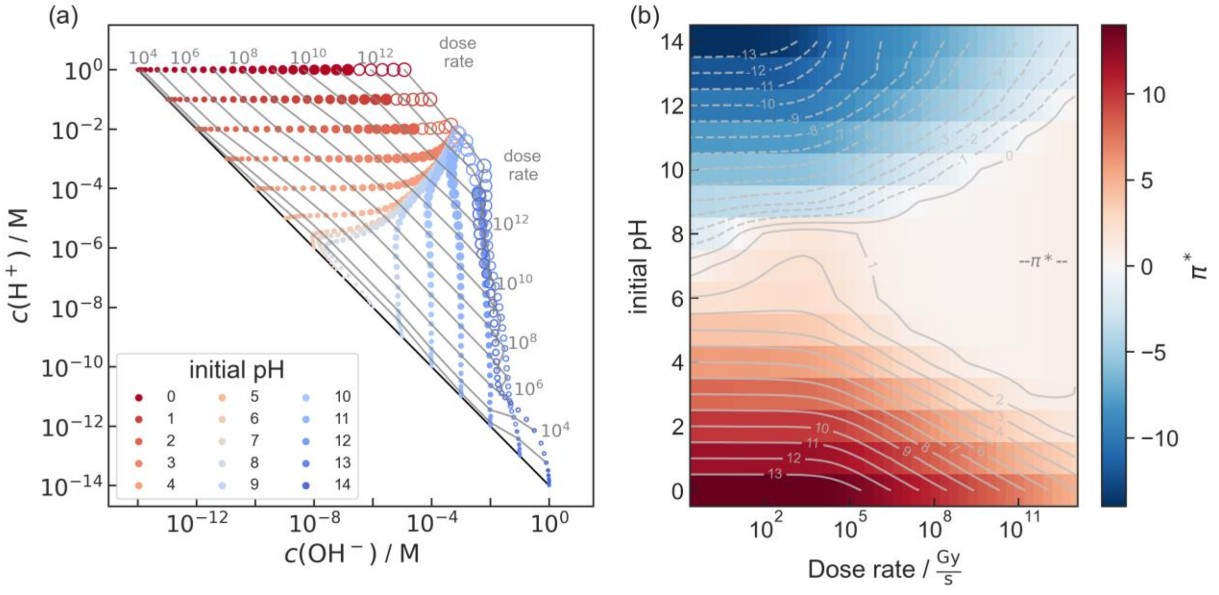

Figure S5: Acid-base chemistry of neat, aerated water as a function of dose rate of incident X-ray radiation and the initial pH value. (a) Concentrations of H^+ and OH^- in the steady state. Each dot represents the steady state concentration of a simulation, while its size is a measure of the dose rate. Dose rate (grey numbers) is given in $Gy \cdot s^{-1}$ and indicated by contour lines. The black diagonal line corresponds to water under equilibrium conditions ($K_W = 10^{-14}$ M^2) without irradiation. Empty dots represent steady states, where the concentration of water is below 99% of the unirradiated solution. (b) π^* (color map and grey contour lines) as function of initial pH and dose rate. The equivalent plots for electron beam irradiation are shown in Figure 3.

S1.5 K_W^* FOR E-BEAM AND X-RAY IRRADIATION

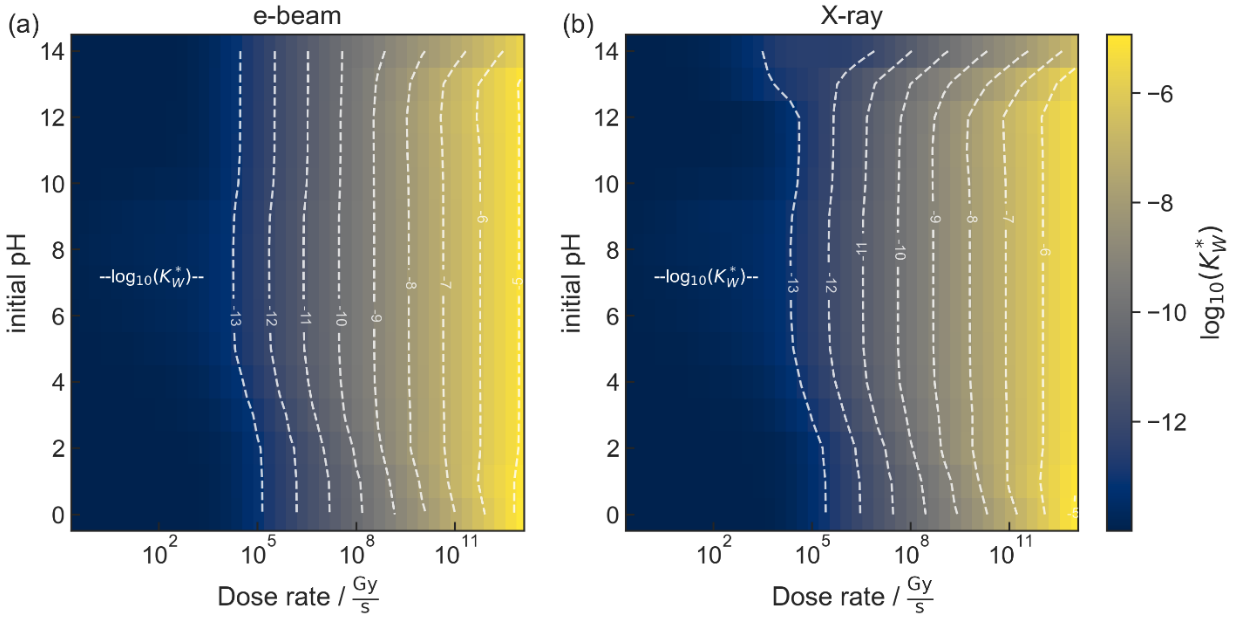

Figure S6: K_W^* depending on dose rate and initial pH for (a) electrons and (b) X-rays.

S1.6 DIFFERENT ADDITIVES

In Figure S7 the concentrations of H^+ and OH^- for initial concentrations of the anions Cl^-, Br^- and NO_3^- of 1 mM as well as 10 mM are compared against pure water (blue).

Figure S8 displays the evolution of the radiolytic ion product relative to the respective value of pure water.

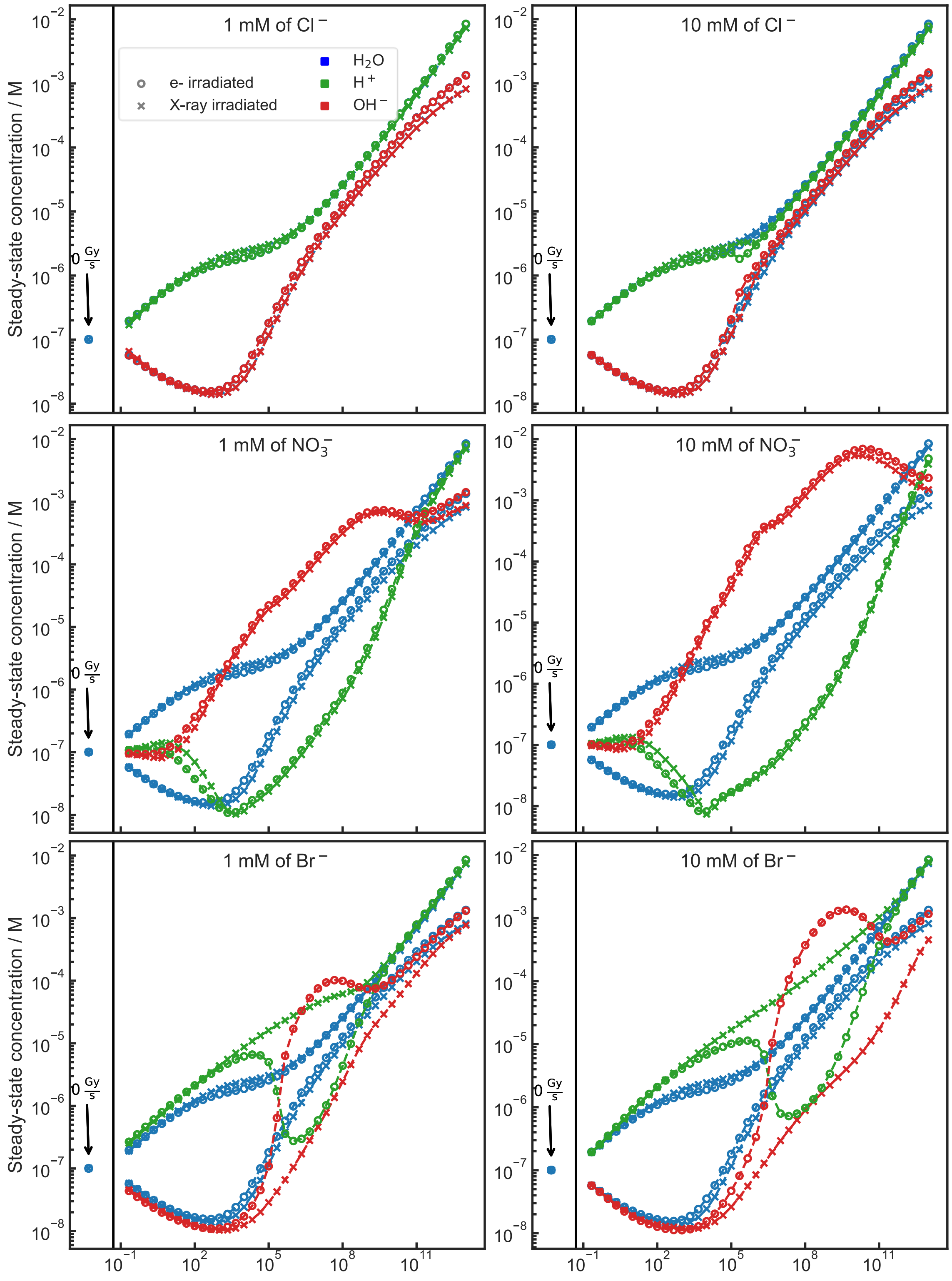

Figure S7: Steady-state concentrations of c(H^+) and c(OH^-) in both, pure, aerated water, and aqueous solutions containing either 1 mM (left) or 10 mM (right) Cl^-, Br^- or NO_3^- ions as functions of the dose rate.

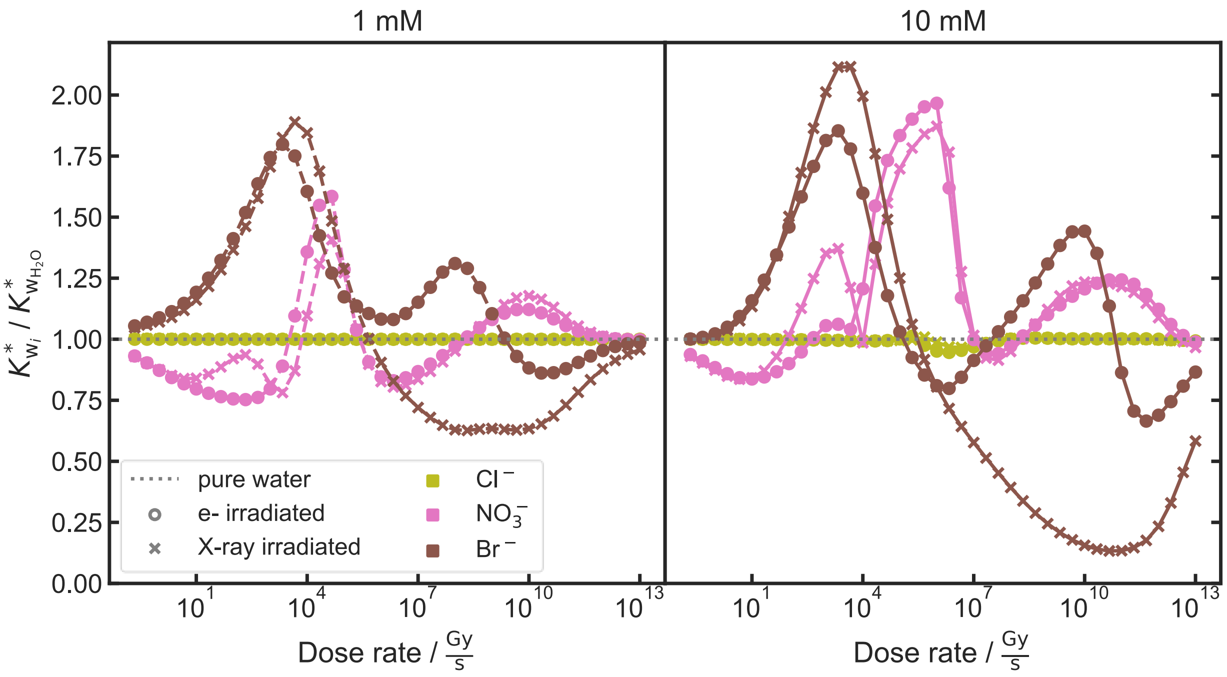

Figure S8: Relation of the ion product K_W^* to the radiolytic ion product of aerated water for aqueous solutions of 1 mM (left) and 10 mM (right). The colors denote the different ionic solutes, whereas the symbol relates to the used G-values.

S1.7 IMPACT OF ALKALI METALS ON THE ACIDITY UNDER IRRADIATION

Albeit the standard potentials of Li^+ is slightly higher than the reductive potential of solvated electrons (see main manuscript), the difference is close to thermal energy. To demonstrate that its impact is negligible for the discussion within this work, we simulated an extreme case scenario, in which we assume the reactivity of Li^+ to be similar to the one of Na^+. For the latter, Tesler and Schindewolf[5] measured a reduction by solvated electrons. They reported the reaction:

$$Na^+ + e_h^- \rightarrow Na \qquad \text{with a rate constant of } 2\cdot10^4\,(Ms)^{-1}$$

As decay, the reaction with H_2O was given within the same manuscript as:

$$2\,Na + 2\,H_2O \rightarrow H_2 + 2\,Na^+ + 2\,OH^- \qquad \text{with a rate constant of } 1.5\cdot10^9\,(Ms)^{-1}$$

Particularly the latter reaction has the potential to alter the acidity under irradiation. However, to simulate elementary steps only, the latter reaction was considered in the form of

$$Na + H_2O \rightarrow H + Na^+ + OH^- \qquad \text{with a rate constant of } 1.5\cdot10^9\,(Ms)^{-1}$$

because the recombination of 2 H → H_2 (Reaction 34 in Table S2) is more than five times faster than the value given here, so that the oxidation of Na was assumed to be rate-determining.

By incorporating these proposed reactions (Table S6) we simulate the evolution of H^+ and OH^- steady state concentrations of 10 mM solutions of pure Na^+, NaBr, NaCl, and $NaNO_3$ under 300 keV electron irradiation (Figure S9). It is evident that Na^+ does not alter the obtained concentrations when considered as a hypothetical stand-alone reactant to pure water. This does not

change notably when more realistic scenarios (NaBr, NaCl, $NaNO_3$) are regarded. Consequently, a change of Li^+ is likely to be negligible throughout all simulations within this manuscript.

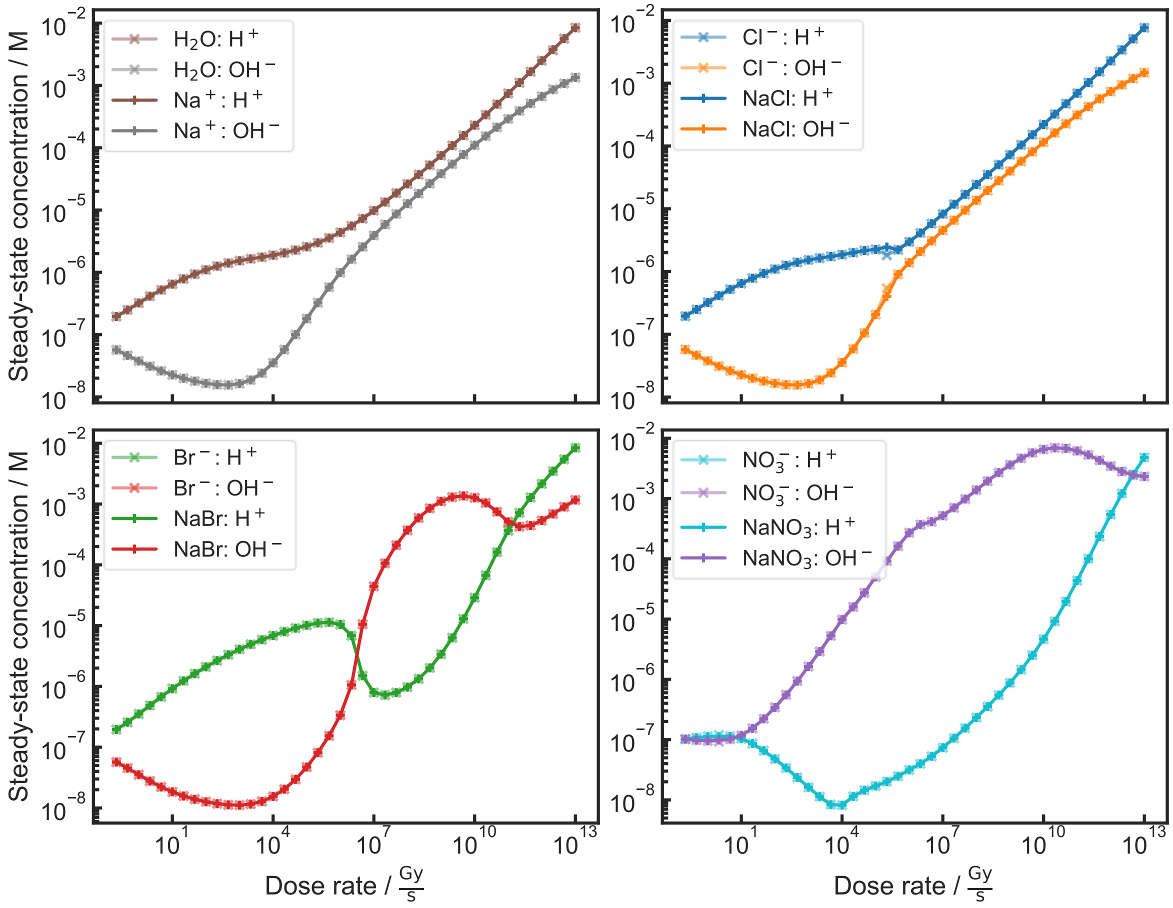

Figure S9: Steady-state evolutions of $c(H^+)$ and $c(OH^-)$ in pure water, and 10 mM Cl^-, Br^-, or NO_3^--containing solutions with and without additional 10 mM Na^+ present.

S1.8 KINETIC MODELS

The following section comprises the reaction sets utilized for simulations shown in this work in tabular and graph network[6,7] format. The latter emphasizes the fundamental difference between irradiated and non-irradiated solutions.

The equilibrium chemistry of pure water is fully described by Equation (1) and shown in Figure 1, the reaction interplay is fully described in (a), while the generation of reactive species by irradiation (Eq. (5)) triggers a reaction cascade comprising 83 reactions and 17 species (b). A tabular representation is shown in Table S2. In addition, the chlorine set comprises 89 reactions and 19 new species (Table S3, Figure S10). Both are a subset of the reaction set used for aqueous $HAuCl_4$ solutions introduced earlier[6].

Br^- containing solutions were described by 52 additional reactions and 10 additional species (Table S4, Figure S11)[8–15]. NO_3^--solutions were simulated using a reaction set of 18 additional species distributed over 73 reactions (Table S5, Figure S12)[12,16–24].

Table S2: Kinetic model for irradiation of neat, aerated water used in this work. k describes the kinetic constant in units of $mol^{-n+1}\,L^{3(n-1)}\,s^{-1}$, where n denotes the reaction order.

	Reaction			*k*	Source
1	H_2O	→	$H^+ + OH^-$	$2.599 \cdot 10^{-5}$	[25]
2	$H^+ + OH^-$	→	H_2O	$1.43 \cdot 10^{11}$	[25]
3	H_2O_2	→	$H^+ + HO_2^-$	$1.119 \cdot 10^{-1}$	[3]
4	$H^+ + HO_2^-$	→	H_2O_2	$5 \cdot 10^{10}$	[3]
5	$H_2O_2 + OH^-$	→	$HO_2^- + H_2O$	$1.3 \cdot 10^{10}$	[3]
6	$HO_2^- + H_2O$	→	$H_2O_2 + OH^-$	$5.82 \cdot 10^{7}$	[3]
7	$e_h^- + H_2O$	→	$H + OH^-$	$1.9 \cdot 10^{1}$	[3]
8	$H + OH^-$	→	$e_h^- + H_2O$	$2.2 \cdot 10^{7}$	[3]
9	H	→	$e_h^- + H^+$	$3.9 \cdot 10^{0}$	[3]
10	$e_h^- + H^+$	→	H	$2.3 \cdot 10^{10}$	[3]
11	$OH + OH^-$	→	$O^- + H_2O$	$1.3 \cdot 10^{10}$	[3]
12	$O^- + H_2O$	→	$OH + OH^-$	$1 \cdot 10^{8}$	[3]
13	OH	→	$O^- + H^+$	$1.259 \cdot 10^{-1}$	[3]
14	$O^- + H^+$	→	OH	$1 \cdot 10^{11}$	[3]
15	HO_2	→	$O_2^- + H^+$	$1.346 \cdot 10^{6}$	[3]
16	$O_2^- + H^+$	→	HO_2	$5 \cdot 10^{10}$	[3]
17	$HO_2 + OH^-$	→	$O_2^- + H_2O$	$5 \cdot 10^{10}$	[3]
18	$O_2^- + H_2O$	→	$HO_2 + OH^-$	$1.862 \cdot 10^{1}$	[3]
19	$e_h^- + OH$	→	OH^-	$3 \cdot 10^{10}$	[3]
20	$e_h^- + H_2O_2$	→	$OH + OH^-$	$1.1 \cdot 10^{10}$	[3]
21	$e_h^- + O_2^- + H_2O$	→	$HO_2^- + OH^-$	$1.3 \cdot 10^{10}$	[3]
22	$e_h^- + HO_2$	→	HO_2^-	$2 \cdot 10^{10}$	[3]
23	$e_h^- + O_2$	→	O_2^-	$1.9 \cdot 10^{10}$	[3]
24	$2\,e_h^- + 2\,H_2O$	→	$H_2 + 2\,OH^-$	$5.5 \cdot 10^{9}$	[3]
25	$e_h^- + H + H_2O$	→	$H_2 + OH^-$	$2.5 \cdot 10^{10}$	[3]
26	$e_h^- + HO_2^-$	→	$O^- + OH^-$	$3.5 \cdot 10^{9}$	[3]
27	$e_h^- + O^- + H_2O$	→	$OH^- + OH^-$	$2.2 \cdot 10^{10}$	[3]
28	$e_h^- + O_3^- + H_2O$	→	$O_2 + OH^- + OH^-$	$1.6 \cdot 10^{10}$	[3]
29	$e_h^- + O_3$	→	O_3^-	$3.6 \cdot 10^{10}$	[3]
30	$H + H_2O$	→	$H_2 + OH$	$1.1 \cdot 10^{1}$	[3]
31	$H + O^-$	→	OH^-	$1 \cdot 10^{10}$	[3]
32	$H + HO_2^-$	→	$OH + OH^-$	$9 \cdot 10^{7}$	[3]

	Reaction			k	Source
33	$H + O_3^-$	→	$OH^- + O_2$	$1 \cdot 10^{10}$	[3]
34	$2\,H$	→	H_2	$7.8 \cdot 10^{9}$	[3]
35	$H + OH$	→	H_2O	$7 \cdot 10^{9}$	[3]
36	$H + H_2O_2$	→	$OH + H_2O$	$9 \cdot 10^{7}$	[3]
37	$H + O_2$	→	HO_2	$2.1 \cdot 10^{10}$	[3]
38	$H + HO_2$	→	H_2O_2	$1.8 \cdot 10^{10}$	[3]
39	$H + O_2^-$	→	HO_2^-	$1.8 \cdot 10^{10}$	[3]
40	$H + O_3$	→	HO_3	$3.8 \cdot 10^{10}$	[3]
41	$2\,OH$	→	H_2O_2	$3.6 \cdot 10^{9}$	[3]
42	$OH + HO_2$	→	$H_2O + O_2$	$6 \cdot 10^{9}$	[3]
43	$OH + O_2^-$	→	$OH^- + O_2$	$8.2 \cdot 10^{9}$	[3]
44	$OH + H_2$	→	$H + H_2O$	$4.3 \cdot 10^{7}$	[3]
45	$OH + H_2O_2$	→	$HO_2 + H_2O$	$2.7 \cdot 10^{7}$	[3]
46	$OH + O^-$	→	HO_2^-	$2.5 \cdot 10^{10}$	[3]
47	$OH + HO_2^-$	→	$HO_2 + OH^-$	$7.5 \cdot 10^{9}$	[3]
48	$OH + O_3^-$	→	$O_3 + OH^-$	$2.6 \cdot 10^{9}$	[3]
49	$OH + O_3^-$	→	$2\,O_2^- + H^+$	$6 \cdot 10^{9}$	[3]
50	$OH + O_3$	→	$HO_2 + O_2$	$1.1 \cdot 10^{8}$	[3]
51	$HO_2 + O_2^-$	→	$HO_2^- + O_2$	$8 \cdot 10^{7}$	[3]
52	$HO_2 + HO_2$	→	$H_2O_2 + O_2$	$7 \cdot 10^{5}$	[3]
53	$HO_2 + O^-$	→	$O_2 + OH^-$	$6 \cdot 10^{9}$	[3]
54	$HO_2 + H_2O_2$	→	$OH + O_2 + H_2O$	$5 \cdot 10^{-1}$	[3]
55	$HO_2 + HO_2^-$	→	$OH + O_2 + OH^-$	$5 \cdot 10^{-1}$	[3]
56	$HO_2 + O_3^-$	→	$O_2 + O_2 + OH^-$	$6 \cdot 10^{9}$	[3]
57	$HO_2 + O_3$	→	$HO_3 + O_2$	$5 \cdot 10^{8}$	[3]
58	$2\,O_2^- + 2\,H_2O$	→	$H_2O_2 + O_2 + 2\,OH^-$	$1 \cdot 10^{2}$	[3]
59	$O_2^- + O^- + H_2O$	→	$O_2 + 2\,OH^-$	$6 \cdot 10^{8}$	[3]
60	$O_2^- + H_2O_2$	→	$OH + O_2 + OH^-$	$1.3 \cdot 10^{-1}$	[3]
61	$O_2^- + HO_2^-$	→	$O^- + O_2 + OH^-$	$1.3 \cdot 10^{-1}$	[3]
62	$O_2^- + O_3^- + H_2O$	→	$O_2 + O_2 + 2\,OH^-$	$1 \cdot 10^{4}$	[3]
63	$O_2^- + O_3$	→	$O_3^- + O_2$	$1.5 \cdot 10^{9}$	[3]
64	$2\,O^- + H_2O$	→	$HO_2^- + OH^-$	$1 \cdot 10^{9}$	[3]
65	$O^- + O_2$	→	O_3^-	$3.6 \cdot 10^{9}$	[3]
66	$O^- + H_2$	→	$H + OH^-$	$8 \cdot 10^{7}$	[3]

	Reaction			k	Source
67	$O^- + H_2O_2$	→	$O_2^- + H_2O$	$5 \cdot 10^{8}$	3
68	$O^- + HO_2^-$	→	$O_2^- + OH^-$	$4 \cdot 10^{8}$	3
69	$O^- + O_3^-$	→	$O_2^- + O_2^-$	$7 \cdot 10^{8}$	3
70	$O^- + O_3$	→	$O_2^- + O_2$	$5 \cdot 10^{9}$	3
71	O_3^-	→	$O_2 + O^-$	$3.3 \cdot 10^{3}$	3
72	$O_3^- + H^+$	→	$O_2 + OH$	$9 \cdot 10^{10}$	3
73	HO_3	→	$O_2 + OH$	$1.1 \cdot 10^{5}$	3
74	H_2O_2	→	$H_2O + O$	$1 \cdot 10^{-3}$	25
75	$2\ O$	→	O_2	$1 \cdot 10^{9}$	25
76	O_3	→	$O_2 + O$	$3 \cdot 10^{-6}$	26
77	$2\ O_3^- + H_2O$	→	$OH^- + HO_2^- + 2\ O_2$	$1 \cdot 10^{4}$	27
78	$2\ HO_3$	→	$H_2O_2 + 2\ O_2$	$5 \cdot 10^{9}$	27
79	$O_3 + OH^-$	→	$HO_2^- + O_2$	$1 \cdot 10^{2}$	27
80	$O_2 + O$	→	O_3	$4 \cdot 10^{9}$	28
81	$H_2O_2 + O$	→	$OH + HO_2$	$1.6 \cdot 10^{9}$	29
82	$O + HO_2^-$	→	$OH + O_2^-$	$5.3 \cdot 10^{9}$	29
83	$O + OH^-$	→	HO_2^-	$4.2 \cdot 10^{8}$	29

Table S3: Kinetic model used to describe the radiolysis of Cl^--containing aqueous solutions. Here, k denotes the respective kinetic constant in units of $mol^{-n+1}\ L^{3(n-1)}\ s^{-1}$, where n denotes the reaction order. Please refer to Table S2 for the first 83 reactions.

	Reaction			k	Source
84	$OH + Cl^-$	→	$ClOH^-$	$4.3 \cdot 10^{9}$	25
85	$OH + HClO$	→	$ClO + H_2O$	$9 \cdot 10^{9}$	25
86	$OH + ClO_2^- + H^+$	→	$ClO_2 + H_2O$	$6.3 \cdot 10^{9}$	25
87	$e_h^- + Cl$	→	Cl^-	$1 \cdot 10^{10}$	25
88	$e_h^- + Cl_2^-$	→	$2\ Cl^-$	$1 \cdot 10^{10}$	25
89	$e_h^- + ClOH^-$	→	$Cl^- + OH^-$	$1 \cdot 10^{10}$	25
90	$e_h^- + HClO$	→	$ClOH^-$	$5.3 \cdot 10^{10}$	25
91	$e_h^- + Cl_2$	→	Cl_2^-	$1 \cdot 10^{10}$	25
92	$e_h^- + Cl_3^-$	→	$Cl_2^- + Cl^-$	$1 \cdot 10^{10}$	25
93	$e_h^- + ClO_2^- + H^+$	→	$ClO + OH^-$	$4.5 \cdot 10^{10}$	25
94	$e_h^- + ClO_3^- + H^+$	→	$ClO_2 + OH^-$	$1 \cdot 10^{10}$	30
95	$H + Cl$	→	$Cl^- + H^+$	$1 \cdot 10^{10}$	25
96	$H + Cl_2^-$	→	$2\ Cl^- + H^+$	$8 \cdot 10^{9}$	25

	Reaction			k	Source
97	$H + ClOH^-$	→	$Cl^- + H_2O$	$1 \cdot 10^{10}$	[25]
98	$H + Cl_2$	→	$Cl_2^- + H^+$	$7 \cdot 10^{9}$	[25]
99	$H + HClO$	→	$ClOH^- + H^+$	$1 \cdot 10^{10}$	[25]
100	$H + Cl_3^-$	→	$Cl_2^- + Cl^- + H^+$	$1 \cdot 10^{10}$	[25]
101	$HO_2 + Cl_2^-$	→	$Cl^- + HCl + O_2$	$4 \cdot 10^{9}$	[25]
102	HCl	→	$Cl^- + H^+$	$5 \cdot 10^{5}$	[25]
103	$Cl^- + H^+$	→	HCl	$6.29 \cdot 10^{-1}$	[25,31]
104	$HO_2 + Cl_2$	→	$Cl_2^- + O_2 + H^+$	$1 \cdot 10^{9}$	[25]
105	$HO_2 + Cl_3^-$	→	$Cl_2^- + HCl + O_2$	$1 \cdot 10^{9}$	[25]
106	$O_2^- + Cl_2^-$	→	$2\ Cl^- + O_2$	$1.2 \cdot 10^{10}$	[25]
107	$O_2^- + HClO$	→	$ClOH^- + O_2$	$7.5 \cdot 10^{6}$	[25]
108	$H_2O_2 + Cl_2^-$	→	$2\ HCl + O_2^-$	$1.4 \cdot 10^{5}$	[25]
109	$H_2O_2 + Cl_2$	→	$HO_2 + Cl_2^- + H^+$	$1.9 \cdot 10^{2}$	[25]
110	$H_2O_2 + HClO$	→	$HCl + H_2O + O_2$	$1.7 \cdot 10^{5}$	[25]
111	$OH^- + Cl_2^-$	→	$ClOH^- + Cl^-$	$7.3 \cdot 10^{6}$	[25]
112	$OH^- + Cl_2$	→	$HClO + Cl^-$	$6 \cdot 10^{8}$	[25]
113	$H^+ + ClOH^-$	→	$Cl + H_2O$	$2.1 \cdot 10^{10}$	[25]
114	$H_2O + Cl_2O_2$	→	$HClO + ClO_2^- + H^+$	$1 \cdot 10^{4}$	[27]
115	$H_2O + Cl_2O$	→	$2\ HClO$	$1 \cdot 10^{2}$	[25]
116	$H_2O + Cl_2O_4$	→	$ClO_2^- + ClO_3^- + 2\ H^+$	$1 \cdot 10^{2}$	[25]
117	$H_2O + Cl_2O_4$	→	$HClO + HCl + O_4$	$1 \cdot 10^{2}$	[25]
118	O_4	→	$2\ O_2$	$1 \cdot 10^{5}$	[25]
119	$Cl^- + Cl$	→	Cl_2^-	$2.1 \cdot 10^{10}$	[25]
120	$Cl^- + ClOH^-$	→	$Cl_2^- + OH^-$	$9 \cdot 10^{4}$	[25]
121	$Cl^- + HClO$	→	$Cl_2 + OH^-$	$1 \cdot 10^{1}$	[30]
122	$Cl^- + Cl_2$	→	Cl_3^-	$1 \cdot 10^{4}$	[25]
123	$ClOH^-$	→	$OH + Cl^-$	$6.1 \cdot 10^{9}$	[25]
124	Cl_2^-	→	$Cl + Cl^-$	$1.1 \cdot 10^{5}$	[25]
125	$2\ Cl_2^-$	→	$Cl_3^- + Cl^-$	$7 \cdot 10^{9}$	[25]
126	Cl_3^-	→	$Cl_2 + Cl^-$	$5 \cdot 10^{4}$	[25]
127	$2\ ClO$	→	Cl_2O_2	$1.5 \cdot 10^{10}$	[25]
128	$2\ ClO_2$	→	Cl_2O_4	$1 \cdot 10^{2}$	[25]

	Reaction			k	Source
129	$Cl_2O_2 + ClO_2^-$	→	$ClO_3^- + Cl_2O$	$1 \cdot 10^{2}$	25
130	$2\ HClO$	→	$Cl^- + ClO_2^-$ $+ 2\ H^+$	$6 \cdot 10^{-9}$	25
131	$ClO_2^- + HClO$	→	$Cl^- + ClO_3^- + H^+$	$9 \cdot 10^{-7}$	25
132	$2\ HClO$	→	$O_2 + 2\ HCl$	$3 \cdot 10^{-10}$	25
133	$HClO + Cl^- + H^+$	→	$Cl_2 + H_2O$	$9 \cdot 10^{3}$	25
134	$Cl_2 + H_2O$	→	$HClO + Cl^- + H^+$	$1.5 \cdot 10^{1}$	25
135	$Cl_2^- + H_2$	→	$H + HCl + Cl^-$	$4.3 \cdot 10^{5}$	25
136	$2\ Cl$	→	Cl_2	$8.8 \cdot 10^{7}$	32
137	$ClO_2 + O_3$	→	$O_2 + ClO_3$	$1.1 \cdot 10^{3}$	33
138	$ClO_2 + OH$	→	$ClO_3^- + H^+$	$4 \cdot 10^{9}$	33
139	$ClO_2 + O^-$	→	ClO_3^-	$2.7 \cdot 10^{9}$	33
140	$ClO_2 + O_3^-$	→	$O_2 + ClO_3^-$	$1.8 \cdot 10^{5}$	33
141	$ClO_2 + O_3^-$	→	$O_3 + ClO_2^-$	$1.8 \cdot 10^{5}$	33
142	$ClO_2^- + O_3$	→	$O_3^- + ClO_2$	$4 \cdot 10^{6}$	33
143	ClO_2	→	$O_2 + Cl$	$6.7 \cdot 10^{9}$	34
144	$HClO$	→	$H^+ + ClO^-$	$2 \cdot 10^{3}$	27
145	$H^+ + ClO^-$	→	$HClO$	$5 \cdot 10^{10}$	27
146	$HClO_2$	→	$H^+ + ClO_2^-$	$9.53 \cdot 10^{8}$	27
147	$H^+ + ClO_2^-$	→	$HClO_2$	$5 \cdot 10^{10}$	27
148	$Cl + O_3^-$	→	$Cl^- + O_3$	$1 \cdot 10^{9}$	27
149	$ClO + O_3^-$	→	$ClO^- + O_3$	$1 \cdot 10^{9}$	27
150	$Cl_2^- + ClO_2$	→	$Cl_2O_2 + Cl^-$	$1 \cdot 10^{9}$	27
151	$Cl + ClO_2$	→	Cl_2O_2	$1 \cdot 10^{9}$	27
152	$ClO + ClO_2^-$	→	$ClO^- + ClO_2$	$9.4 \cdot 10^{8}$	27
153	$ClO^- + O^- + H^+$	→	$ClO + OH^-$	$2.3 \cdot 10^{8}$	27
154	$Cl^- + H_2O_2$	→	$ClO^- + H_2O$	$1.8 \cdot 10^{-9}$	27
155	$Cl^- + H_2O_2 + H^+$	→	$HClO + H_2O$	$8.3 \cdot 10^{-7}$	27
156	$ClO^- + H_2O_2$	→	$Cl^- + O_2 + H_2O$	$3.4 \cdot 10^{3}$	27
157	$HClO + HO_2^-$	→	$Cl^- + O_2 + H_2O$	$4.4 \cdot 10^{7}$	27
158	$Cl_2 + HO_2^-$	→	$2\ Cl^- + O_2 + H^+$	$1.1 \cdot 10^{8}$	27
159	$Cl + H_2O_2$	→	$Cl^- + H^+ + HO_2$	$2 \cdot 10^{9}$	27
160	$Cl + HO_2$	→	$Cl^- + H^+ + O_2$	$3.1 \cdot 10^{9}$	27
161	$Cl + OH^-$	→	$ClOH^-$	$1.8 \cdot 10^{10}$	27

	Reaction			k	Source
162	$ClO_2 + H_2O_2$	→	$ClO_2^- + H^+ + HO_2$	$4 \cdot 10^{0}$	[27]
163	$ClO_2 + HO_2^-$	→	$ClO_2^- + HO_2$	$1.3 \cdot 10^{5}$	[27]
164	$ClO_2 + HO_2$	→	$ClO_2^- + H^+ + O_2$	$1 \cdot 10^{6}$	[27]
165	$ClO_2 + O_2^-$	→	$ClO_2^- + O_2$	$3 \cdot 10^{9}$	[27]
166	$ClO_2^- + O_2^-$	→	$ClO^- + O^- + O_2$	$4 \cdot 10^{1}$	[27]
167	$ClO + ClO_2$	→	Cl_2O_3	$7.4 \cdot 10^{9}$	[27]
168	$ClO + ClO_3$	→	Cl_2O_4	$7.4 \cdot 10^{9}$	[27]
169	$Cl_2O_2 + OH^-$	→	$Cl^- + ClO_3^- + H^+$	$1 \cdot 10^{10}$	[27]
170	$Cl_2O_3 + H_2O$	→	$HClO + ClO_3^- + H^+$	$1 \cdot 10^{4}$	[27]
171	$ClOH^-$	→	$Cl + OH^-$	$2.3 \cdot 10^{1}$	[27]
172	$Cl + H_2O$	→	$ClOH^- + H^+$	$1.8 \cdot 10^{5}$	[27]
173	$Cl_2^- + O_3$	→	$ClO + Cl^- + O_2$	$9 \cdot 10^{7}$	[27]

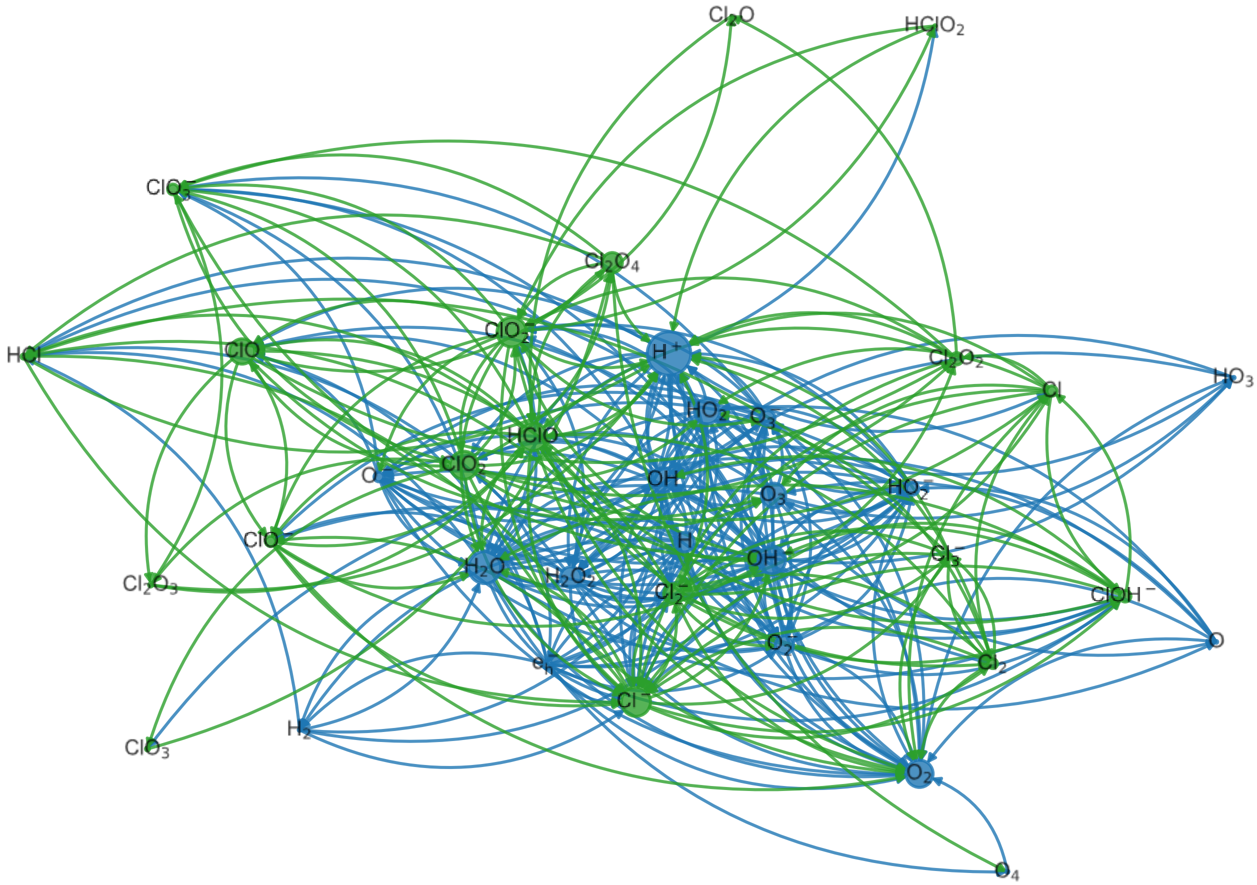

Figure S10: Graph representation of the kinetic model of Cl^--containing aqueous solutions. Tabular representation is found in Table S3.

Table S4: Kinetic model used to describe the radiolysis of Br^--containing aqueous solutions. Here, k denotes the respective kinetic constant in units of $mol^{-n+1}\ L^{3(n-1)}\ s^{-1}$, where n denotes the respective reaction order. Please refer to Table S2 for the first 83 reactions.

	Reaction			k	Source
84	$Br^- + OH$	→	$BrOH^-$	$1.1 \cdot 10^{10}$	8
85	$BrOH^-$	→	$Br^- + OH$	$3.3 \cdot 10^{7}$	8
86	$BrOH^- + H^+$	→	$Br + H_2O$	$4.4 \cdot 10^{10}$	8
87	$BrOH^-$	→	$Br + OH^-$	$4.2 \cdot 10^{6}$	8
88	$Br + OH^-$	→	$BrOH^-$	$1.3 \cdot 10^{10}$	8
89	$Br + Br^-$	→	Br_2^-	$1.2 \cdot 10^{10}$	8
90	Br_2^-	→	$Br + Br^-$	$1.9 \cdot 10^{4}$	8
91	$2\ Br_2^-$	→	$Br_3^- + Br^-$	$2.4 \cdot 10^{9}$	8
92	$Br + Br_2^-$	→	Br_3^-	$5 \cdot 10^{9}$	8
93	$Br_2 + Br^-$	→	Br_3^-	$1.6 \cdot 10^{8}$	8
94	Br_3^-	→	$Br_2 + Br^-$	$1 \cdot 10^{7}$	8
95	$2\ Br$	→	Br_2	$5 \cdot 10^{9}$	8
96	$Br + e_h^-$	→	Br^-	$1 \cdot 10^{10}$	8
97	$Br_2^- + e_h^-$	→	$2\ Br^-$	$1.3 \cdot 10^{10}$	8
98	$Br_3^- + e_h^-$	→	$Br_2^- + Br^-$	$2.7 \cdot 10^{10}$	8
99	$H + Br$	→	$H^+ + Br^-$	$1 \cdot 10^{10}$	8
100	$Br_2^- + H$	→	$2\ Br^- + H^+$	$1.4 \cdot 10^{10}$	8
101	$Br_3^- + H$	→	$Br_2^- + Br^- + H^+$	$1.2 \cdot 10^{10}$	8
102	$Br_2^- + HO_2$	→	$O_2 + H^+ + 2\ Br^-$	$1 \cdot 10^{8}$	8
103	$Br_3^- + HO_2$	→	$Br_2^- + HBr + O_2$	$1 \cdot 10^{7}$	8
104	$BrOH^- + Br^-$	→	$Br_2^- + OH^-$	$1.9 \cdot 10^{8}$	8
105	$Br_2^- + OH^-$	→	$BrOH^- + Br^-$	$2.7 \cdot 10^{6}$	8
106	$Br^- + H$	→	HBr^-	$1.7 \cdot 10^{6}$	8
107	$HBr^- + H^+$	→	$H_2 + Br$	$1.1 \cdot 10^{10}$	8
108	$H^+ + Br^-$	→	HBr	$1 \cdot 10^{4}$	9
109	HBr	→	$H^+ + Br^-$	$1 \cdot 10^{13}$	9
110	$2\ Br_2^-$	→	$Br_2 + 2\ Br^-$	$1.9 \cdot 10^{9}$	10
111	$Br + Br_2^-$	→	$Br_2 + Br^-$	$2 \cdot 10^{9}$	10
112	$Br_2 + e_h^-$	→	Br_2^-	$5.3 \cdot 10^{10}$	11
113	$Br_2 + H$	→	$Br_2^- + H^+$	$1 \cdot 10^{10}$	12
114	$Br^- + O_3$	→	$BrO^- + O_2$	$1.6 \cdot 10^{2}$	13
115	$Br + H_2O$	→	$BrOH^- + H^+$	$1.36 \cdot 10^{0}$	14

116	$Br + H_2O_2$	$\rightarrow$	$O_2^- + Br^- + 2\,H^+$	$4 \cdot 10^9$	10
117	$Br + HO_2$	$\rightarrow$	$H^+ + O_2 + Br^-$	$1 \cdot 10^9$	10
118	$Br_2^- + Br$	$\rightarrow$	$Br_2 + Br^-$	$2 \cdot 10^9$	10
119	$Br_2^- + H_2O_2$	$\rightarrow$	$HO_2 + 2\,Br^- + H^+$	$5 \cdot 10^2$	10
120	$Br_2^- + O_2^-$	$\rightarrow$	$O_2 + 2\,Br^-$	$1.7 \cdot 10^8$	10
121	$Br_2 + HO_2$	$\rightarrow$	$Br_2^- + O_2 + H^+$	$1.1 \cdot 10^8$	10
122	$Br_2 + O_2^-$	$\rightarrow$	$Br_2^- + O_2$	$5.6 \cdot 10^9$	10
123	$Br_2 + H_2O_2$	$\rightarrow$	$2\,HBr + O_2$	$1.3 \cdot 10^3$	10
124	$Br_2 + H_2O$	$\rightarrow$	$HOBr + Br^- + H^+$	$9.7 \cdot 10^1$	10
125	$Br_3^- + O_2^-$	$\rightarrow$	$Br_2^- + Br^- + O_2$	$3.8 \cdot 10^9$	10
126	$BrO^- + H^+$	$\rightarrow$	$HOBr$	$1 \cdot 10^{10}$	9
127	$HOBr$	$\rightarrow$	$H^+ + BrO^-$	$2.3 \cdot 10^1$	9
128	$Br_2^- + OH$	$\rightarrow$	$HOBr + Br^-$	$1 \cdot 10^9$	10
129	$HOBr + Br^- + H^+$	$\rightarrow$	$Br_2 + H_2O$	$5 \cdot 10^9$	10
130	$HOBr + HO_2^-$	$\rightarrow$	$Br^- + H_2O + O_2$	$7.6 \cdot 10^8$	10
131	$HOBr + H_2O_2$	$\rightarrow$	$HBr + H_2O + O_2$	$1.5 \cdot 10^4$	10
132	$HOBr + O_2^-$	$\rightarrow$	$BrOH^- + O_2$	$3.5 \cdot 10^9$	10
133	$BrO^- + H_2O_2$	$\rightarrow$	$Br^- + H_2O + O_2$	$1.2 \cdot 10^6$	10
134	$BrO^- + O_2^- + H_2O$	$\rightarrow$	$Br + 2\,OH^- + O_2$	$1 \cdot 10^2$	10
135	$BrO^- + e_h^-$	$\rightarrow$	$Br^- + O^-$	$1.5 \cdot 10^{10}$	12,15

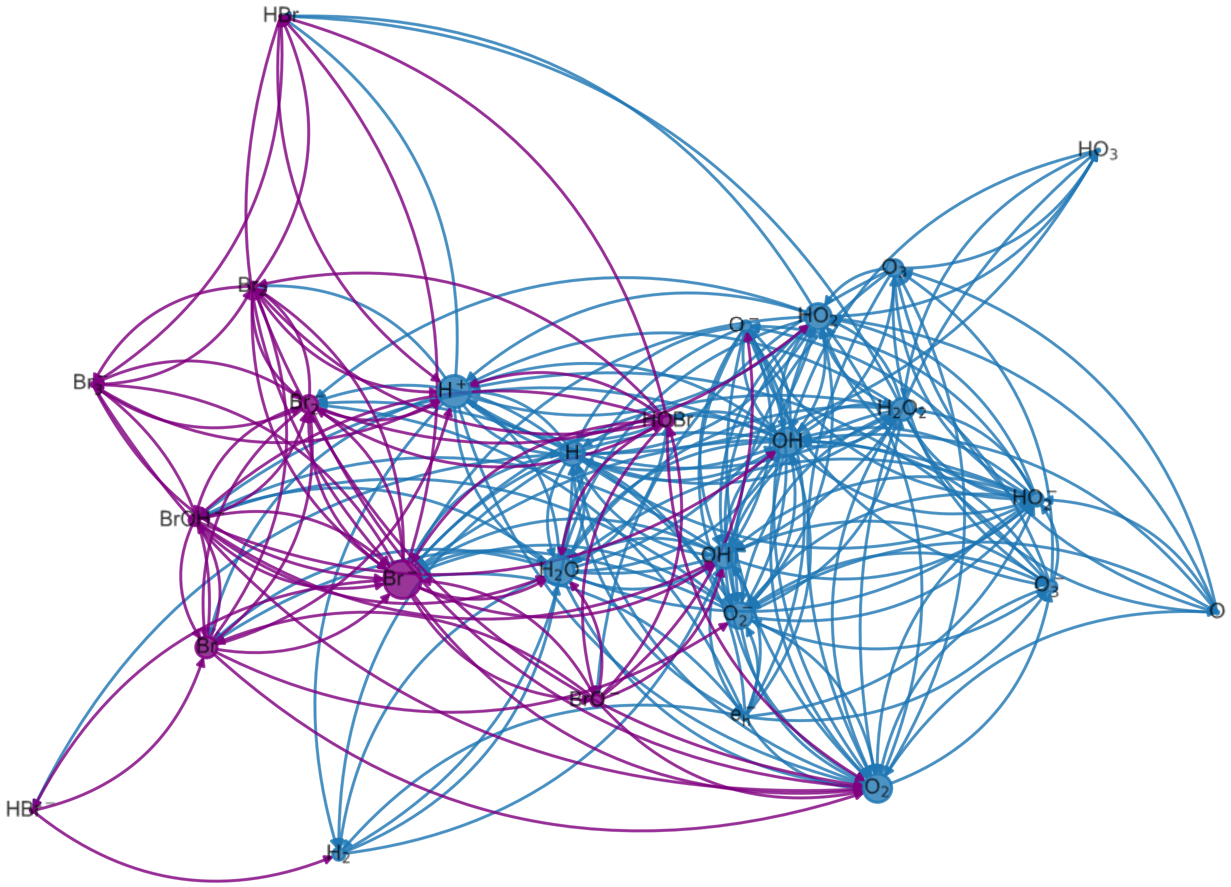

Figure S11: Graph representation of the kinetic model of Br$^-$ -containing aqueous solutions. Tabular representation is found in Table S4.

Table S5: Kinetic model used to describe the radiolysis of NO_3^--containing aqueous solutions. Here, k denotes the respective kinetic constant in units of mol^{-n+1} $L^{3(n-1)}$ s^{-1}, where n denotes the reaction order. Please refer to Table S2 for the first 83 reactions.

	Reaction			k	Source
84	$NO_3^- + e_h^-$	→	NO_3^{2-}	$9.7 \cdot 10^9$	16
85	$NO_3^- + H$	→	HNO_3^-	$5.6 \cdot 10^6$	17
86	$NO_3^- + H^+$	→	HNO_3	$6 \cdot 10^8$	17
87	HNO_3	→	$H^+ + NO_3^-$	$1.46 \cdot 10^{10}$	17
88	$HNO_3 + OH$	→	$NO_3 + H_2O$	$1.9 \cdot 10^7$	17
89	$NO_3^{2-} + OH$	→	$NO_3^- + OH^-$	$3 \cdot 10^9$	17
90	$NO_3^{2-} + H_2O_2$	→	$NO_3^- + OH + OH^-$	$1.6 \cdot 10^8$	17
91	$NO_3^{2-} + O_2$	→	$NO_3^- + O_2^-$	$2.4 \cdot 10^8$	17
92	$NO_3^{2-} + H_2O$	→	$NO_2 + 2\ OH^-$	$1 \cdot 10^3$	17
93	HNO_3^-	→	$NO_3^{2-} + H^+$	$1.6 \cdot 10^3$	17

	Reaction			*k*	Source
94	NO_2 + e_h^-	→	NO_2^-	$1 \cdot 10^{10}$	17
95	NO_2 + OH	→	HOONO	$4.5 \cdot 10^{9}$	17
96	NO_2 + HO_2	→	$HOONO_2$	$1.8 \cdot 10^{9}$	17
97	NO_2 + H	→	HNO_2	$1 \cdot 10^{10}$	17
98	NO_2 + O_2^-	→	O_2NOO^-	$4.5 \cdot 10^{9}$	17
99	2 NO_2	→	N_2O_4	$4.5 \cdot 10^{9}$	17
100	NO_2 + NO_3	→	NO + NO_2 + O_2	$2.41 \cdot 10^{5}$	17
101	NO_2 + NO	→	N_2O_3	$1.1 \cdot 10^{9}$	17
102	NO_2 + O^-	→	$ONOO^-$	$3.5 \cdot 10^{9}$	17
103	N_2O_4	→	2 NO_2	$6 \cdot 10^{3}$	17
104	N_2O_4 + H_2O	→	HNO_2 + HNO_3	$1.8 \cdot 10^{1}$	17
105	HNO_2 + OH	→	NO_2 + H_2O	$2 \cdot 10^{9}$	17
106	HNO_2	→	NO_2^- + H^+	$3 \cdot 10^{7}$	17
107	2 HNO_2	→	NO_2 + NO<br>+ H_2O	$1.34 \cdot 10^{1}$	17
108	HNO_2 + e_h^-	→	HNO_2^-	$4 \cdot 10^{9}$	17
109	HNO_2 + H	→	H_2NO_2	$3.88 \cdot 10^{8}$	18
110	HNO_2 + NO_3	→	NO_2 + HNO_3	$2 \cdot 10^{8}$	17
111	HNO_2 + HNO_3	→	2 NO_2 + H_2O	$6.62 \cdot 10^{3}$	17
112	NO_2^- + H^+	→	HNO_2	$5 \cdot 10^{10}$	17
113	NO_2^- + OH	→	NO_2 + OH^-	$1 \cdot 10^{10}$	17
114	NO_2^- + H	→	HNO_2^-	$1.64 \cdot 10^{9}$	18
115	NO_2^- + O^- + H_2O	→	NO_2 + 2 OH^-	$3.1 \cdot 10^{8}$	17
116	NO_2^- + e_h^-	→	NO_2^{2-}	$4.1 \cdot 10^{9}$	17
117	NO_2^- + NO_3	→	NO_2 + NO_3^-	$4.4 \cdot 10^{9}$	17
118	2 NO_3	→	2 NO_2 + O_2	$1.3 \cdot 10^{5}$	19
119	NO_3 + H_2O_2	→	HNO_3 + HO_2	$7.1 \cdot 10^{6}$	17
120	NO_3 + OH	→	NO_2 + HO_2	$1 \cdot 10^{10}$	17
121	NO_3 + HO_2	→	HNO_3 + O_2	$3 \cdot 10^{9}$	17
122	NO_3 + H_2O	→	HNO_3 + OH	$3 \cdot 10^{2}$	17
123	NO_3 + OH^-	→	NO_3^- + OH	$8.2 \cdot 10^{7}$	17
124	HOONO	→	NO_3^- + H^+	$9 \cdot 10^{-1}$	17
125	HOONO	→	NO_2 + OH	$3.5 \cdot 10^{-1}$	17
126	$HOONO_2$	→	NO_2 + HO_2	$2.6 \cdot 10^{-2}$	17

	Reaction			k	Source
127	$HOONO_2$	→	$HNO_2 + O_2$	$7 \cdot 10^{-4}$	[17]
128	$HOONO + H_2O$	→	$HNO_2 + H_2O_2$	$3 \cdot 10^{2}$	[17]
129	$HOONO_2$	→	$O_2NOO^- + H^+$	$7.1 \cdot 10^{4}$	[17]
130	$HOONO_2 + HNO_2$	→	$2\ HNO_3$	$1.2 \cdot 10^{1}$	[17]
131	O_2NOO^-	→	$NO_2^- + O_2$	$1.35 \cdot 10^{0}$	[17]
132	O_2NOO^-	→	$NO_2 + O_2^-$	$1 \cdot 10^{0}$	[17]
133	$O_2NOO^- + H^+$	→	$HOONO_2$	$5 \cdot 10^{10}$	[17]
134	HNO_2^-	→	$NO + OH^-$	$5 \cdot 10^{3}$	[17]
135	$NO_2^{2-} + H_2O$	→	$NO + 2\ OH^-$	$1.6 \cdot 10^{6}$	[17]
136	$2\ NO + O_2$	→	$2\ NO_2$	$5.9 \cdot 10^{6}$	[17]
137	$NO + OH$	→	$NO_2^- + H^+$	$1 \cdot 10^{10}$	[17]
138	$NO + HO_2$	→	$HOONO$	$3.2 \cdot 10^{9}$	[17]
139	$NO + O_2^-$	→	$ONOO^-$	$5 \cdot 10^{9}$	[17]
140	$ONOO^-$	→	$NO + O_2^-$	$2 \cdot 10^{-2}$	[17]
141	$HOONO + H^+$	→	$HNO_3 + H^+$	$4.3 \cdot 10^{0}$	[17]
142	$ONOO^- + OH$	→	$NO + O_2 + OH^-$	$4.8 \cdot 10^{9}$	[17]
143	N_2O_3	→	$NO + NO_2$	$8.4 \cdot 10^{4}$	[17]
144	$ONOO^- + N_2O_3$	→	$2\ NO_2 + NO_2^-$	$3.1 \cdot 10^{8}$	[17]
145	$N_2O_3 + H_2O$	→	$2\ NO_2^- + 2\ H^+$	$2 \cdot 10^{3}$	[17]
146	$ONOO^- + H^+$	→	$HOONO$	$5 \cdot 10^{10}$	[17]
147	$ONOO^- + NO_2$	→	$NO_2^- + NO_3$	$2.4 \cdot 10^{4}$	[17]
148	$H_2NO_2 + O_2^-$	→	$ONOO^- + H_2O$	$2.3 \cdot 10^{7}$	[12,20]
149	$NO_3^{2-} + H^+$	→	$NO_2 + OH^-$	$2 \cdot 10^{10}$	[12]
150	HNO_3^-	→	$NO_2 + OH^-$	$2 \cdot 10^{5}$	[12]
151	$HNO_2 + H$	→	$H_2O + NO$	$4.5 \cdot 10^{8}$	[21]
152	$HNO_2 + H_2O_2$	→	$NO_3^- + H^+ + H_2O$	$4.6 \cdot 10^{3}$	[22]
153	$NO + NO_2 + H_2O$	→	$2\ HNO_2$	$1.58 \cdot 10^{8}$	[19]
154	$2\ NO_2 + H_2O$	→	$HNO_2 + HNO_3$	$4.8 \cdot 10^{7}$	[19]
155	$HOONO$	→	$ONOO^- + H^+$	$5 \cdot 10^{4}$	[23]
156	$NO_2^- + O_3$	→	$O_2 + NO_3^-$	$3.7 \cdot 10^{5}$	[24]

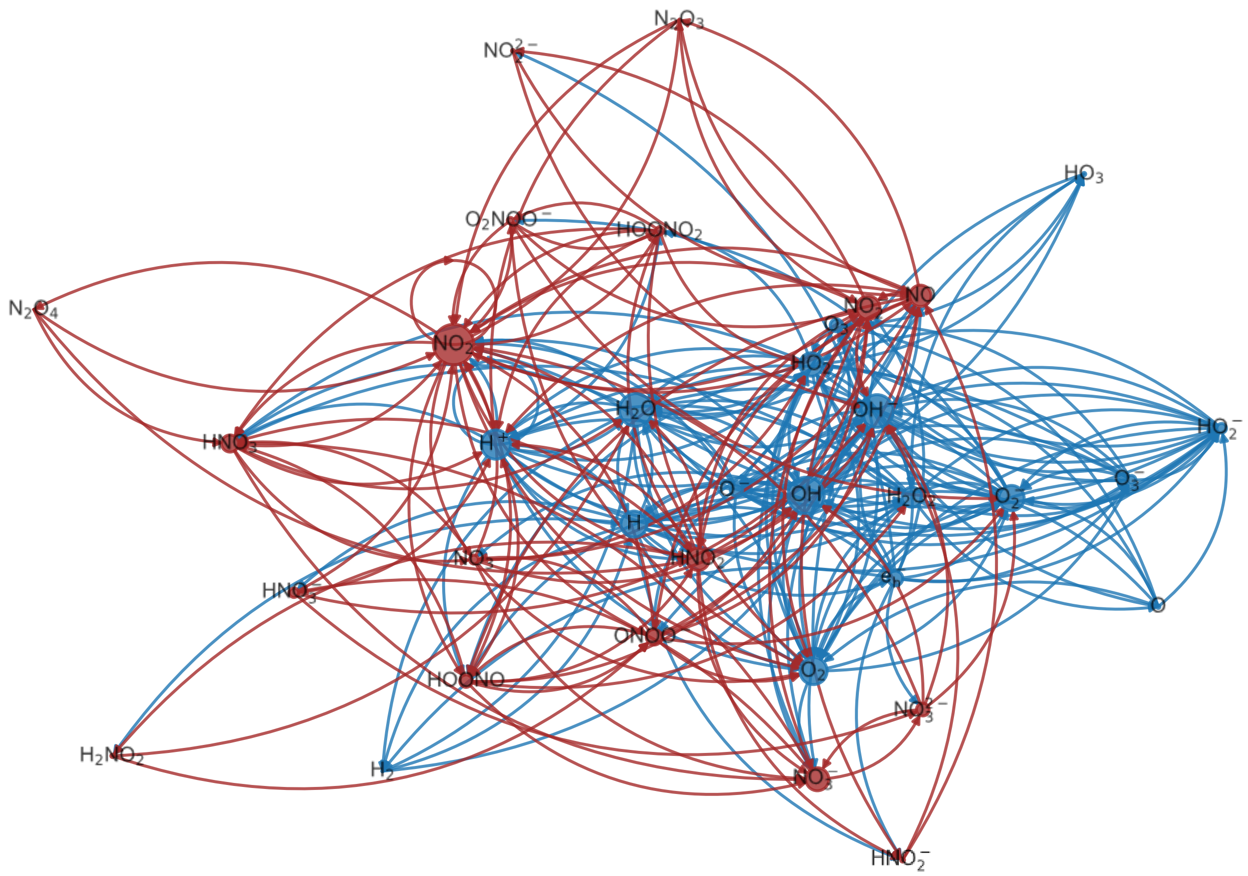

Figure S12: Graph representation of the kinetic model of NO_3^- -containing aqueous solutions. Tabular representation is found in Table S5.

Table S6: Kinetic model used to describe the radiolysis of Na^+-containing aqueous solutions. Here, k denotes the respective kinetic constant in units of $mol^{-n+1}\,L^{3(n-1)}\,s^{-1}$, where n denotes the reaction order. Please refer to Table S2 for the first 83 reactions.

	Reaction			k	Source
84	$Na^+ + e_h^-$	→	Na	$2 \cdot 10^4$	[5]
85	$Na + H_2O$	→	$H + Na^+ + OH^-$	$1.5 \cdot 10^9$	Assumed as rate-determining elementary step, after ref.[5]

Supplementary References

(1) Le Caër, S. Water Radiolysis: Influence of Oxide Surfaces on H_2 Production under Ionizing Radiation. *Water* **2011**, *3*, 235–253.

(2) Buxton, G. V., Sellers, R. M. The radiation chemistry of metal ions in aqueous solution. *Coord. Chem. Rev.* **1977**, *22*, 195–274.

(3) Schneider, N. M., Norton, M. M., Mendel, B. J., Grogan, J. M., Ross, F. M., Bau, H. H. Electron–Water Interactions and Implications for Liquid Cell Electron Microscopy. *J. Phys. Chem. C* **2014**, *118*, 22373–22382.

(4) Pastina, B., LaVerne, J. A. Effect of Molecular Hydrogen on Hydrogen Peroxide in Water Radiolysis. *J. Phys. Chem. A* **2001**, *105*, 9316–9322.

(5) Telser, T., Schindewolf, U. Reaction of hydrated electrons with alkali metal cations in alkaline aqueous solutions. *J. Phys. Chem.* **1986**, *90*, 5378–5382.
(6) Fritsch, B., Zech, T. S., Bruns, M. P., Körner, A., Khadivianazar, S., Wu, M., Zargar Talebi, N., Virtanen, S., Unruh, T., Jank, M. P. M., Spiecker, E., Hutzler, A. Radiolysis-Driven Evolution of Gold Nanostructures – Model Verification by Scale Bridging in situ Liquid-Phase Transmission Electron Microscopy and X-Ray Diffraction. *Adv. Sci.* **2022**, *9*, 2202803.
(7) Holmes, T. D., Rothman, R. H., Zimmerman, W. B. Graph Theory Applied to Plasma Chemical Reaction Engineering. *Plasma Chem. Plasma Process.* **2021**, *41*, 531–557.
(8) El Omar, A. K., Schmidhammer, U., Balcerzyk, A., LaVerne, J., Mostafavi, M. Spur reactions observed by picosecond pulse radiolysis in highly concentrated bromide aqueous solutions. *J. Phys. Chem. A* **2013**, *117*, 2287–2293.
(9) Williams, J. E., Dentener, F. J., van den Berg, A. R. The influence of cloud chemistry on HO_x and NO_x in the moderately polluted marine boundary layer: a 1-D modelling study. *Atmos. Chem. Phys.* **2002**, *2*, 39–54.
(10) Yang, Y., Pignatello, J. J. Participation of the Halogens in Photochemical Reactions in Natural and Treated Waters. *Molecules* **2017**, *22*.
(11) Schwarz, H. A., Gill, P. S. Diffusion-limited solvated electron reactions in ethanol and water. *J. Phys. Chem.* **1977**, *81*, 22–25.
(12) Huie R. E. *NDRL/NIST Solution Kinetics Database on the WEB*: Gaithersburg, MD, 2003.
(13) Haag, W. R., Hoigne, J. Ozonation of bromide-containing waters: kinetics of formation of hypobromous acid and bromate. *Environ. Sci. Technol.* **1983**, *17*, 261–267.
(14) Kläning, U. K., Wolff, T. Laser Flash Photolysis of HClO, ClO^-, HBrO, and BrO^- in Aqueous Solution. Reactions of Cl- and Br-Atoms. *Ber. Bunsenges. Phys. Chem.* **1985**, *89*, 243–245.
(15) Buxton, G. V., Dainton, F. S. The Radiolysis of aqueous Solutions of Oxybromine Compounds; the Spectra and Reactions of BrO and BrO_2. *Proc. R. Soc. Lond. A* **1968**, 427–439.
(16) Buxton, G. V., Greenstock, C. L., Helman, W. P., Ross, A. B. Critical Review of rate constants for reactions of hydrated electrons, hydrogen atoms and hydroxyl radicals (·OH/·O − in Aqueous Solution. *J. Phys. Chem. ref. Data* **1988**, *17*, 513–886.
(17) Horne, G. P., Donoclift, T. A., Sims, H. E., Orr, R. M., Pimblott, S. M. Multi-Scale Modeling of the Gamma Radiolysis of Nitrate Solutions. *J. Phys. Chem. B* **2016**, *120*, 11781–11789.
(18) Mezyk, S. P., Bartels, D. M. Temperature Dependence of Hydrogen Atom Reaction with Nitrate and Nitrite Species in Aqueous Solution. *J. Phys. Chem. A* **1997**, *101*, 6233–6237.
(19) McKenzie, H., MacDonald-Taylor, J., McLachlan, F., Orr, R., Woodhead, D. Modelling of Nitric and Nitrous Acid Chemistry for Solvent Extraction Purposes. *Procedia Chem.* **2016**, *21*, 481–486.
(20) Mikhailova, T. L., Ershov, B. G. The mechanism of nitrite formation in the radiation-induced oxidation of ammonia in aerated aqueous solutions. *Russ. Chem. Bull.* **1993**, *42*, 235–238.
(21) Halpern, J., Rabani, J. Reactivity of Hydrogen Atoms toward Some Cobalt(III) Complexes in Aqueous Solutions 1. *J. Am. Chem. Soc.* **1966**, *88*, 699–704.
(22) Leriche, M. Modeling study of strong acids formation and partitioning in a polluted cloud during wintertime. *J. Geophys. Res.* **2003**, *108*.

(23) Herrmann, H., Ervens, B., Jacobi, H.-W., Wolke, R., Nowacki, P., Zellner, R. CAPRAM2.3: A Chemical Aqueous Phase Radical Mechanism for Tropospheric Chemistry. *J. Atmos. Chem.* **2000**, *36*, 231–284.
(24) Hoigné, J., Bader, H., Haag, W., Staehelin, J. Rate constants of reactions of ozone with organic and inorganic compounds in water—III. Inorganic compounds and radicals. *Water Res.* **1985**, *19*, 993–1004.
(25) Kelm M., Bohnert E. *A kinetic model for the radiolysis of chloride brine, its sensitivity against model parameters and a comparison with experiments: Wissenschaftliche Berichte FZKA 6977*; Karlsruhe: Karlsruhe, 2004.
(26) Sehested, K., Corfitzen, H., Holcman, J., Hart, E. J. Decomposition of ozone in aqueous acetic acid solutions (pH 0-4). *J. Phys. Chem.* **1992**, *96*, 1005–1009.
(27) Levanov, A. V., Isaikina, O. Y. Mechanism and Kinetic Model of Chlorate and Perchlorate Formation during Ozonation of Aqueous Chloride Solutions. *Ind. Eng. Chem. Res.* **2020**, *59*, 14278–14287.
(28) Kläning, U. K., Sehested, K., Wolff, T. Ozone formation in laser flash photolysis of oxoacids and oxoanions of chlorine and bromine. *J. Chem. Soc., Faraday Trans. 1* **1984**, *80*, 2969.
(29) Sauer, M. C., Brown, W. G., Hart, E. J. Oxygen(3P) atom formation by the photolysis of hydrogen peroxide in alkaline aqueous solutions. *J. Phys. Chem.* **1984**, *88*, 1398–1400.
(30) Sunder, S., Christensen, H. Gamma Radiolysis of Water Solutions Relevant to the Nuclear Fuel Waste Management Program. *Nucl. Technol.* **1993**, *104*, 403–417.
(31) Trummal, A., Lipping, L., Kaljurand, I., Koppel, I. A., Leito, I. Acidity of Strong Acids in Water and Dimethyl Sulfoxide. *J. Phys. Chem. A* **2016**, *120*, 3663–3669.
(32) Wu, D., Wong, D., Di Bartolo, B. Evolution of Cl−2 in aqueous NaCl solutions. *J. Photochem.* **1980**, *14*, 303–310.
(33) Klaning, U. K., Sehested, K., Holcman, J. Standard Gibbs energy of formation of the hydroxyl radical in aqueous solution. Rate constants for the reaction chlorite (ClO2-) + ozone .dblarw. ozone(1-) + chlorine dioxide. *J. Phys. Chem.* **1985**, *89*, 760–763.
(34) Dunn, R. C., Simon, J. D. Excited-state photoreactions of chlorine dioxide in water. *J. Am. Chem. Soc.* **1992**, *114*, 4856–4860.
