# Supplementary material for: Tailoring the Acidity of Liquid Media with Ionizing Radiation: Rethinking the Acid–Base Correlation beyond pH
Source: J Phys Chem Lett. 2023 May 11;14(20):4644–51. doi: 10.1021/acs.jpclett.3c00593 (PMC10226122; doi:10.1021/acs.jpclett.3c00593)
Supplement: Supplementary file 2 — jz3c00593_si_002.pdf [file jz3c00593_si_002.pdf]

jz-2023-005937.R1

Name: Peer Review Information for "Tailoring the Acidity of Liquid Media with Ionizing Radiation: Rethinking the Acid-Base Correlation Beyond pH"

First Round of Reviewer Comments

Reviewer: 1

Comments to the Author
In this paper, Fritsch et al reported their study of ionizing radiation induced acidity of liquids. This is a topic that has not been well understood… Nowadays, liquid phase electron microscopy and in-situ X-ray characterization methods have attracted significant interest. An understanding of the radiation induced changes in chemistry, or more specially acidity variation is of great interest. In this paper, the authors used kinetic modeling of radiation chemistry and showed that that pH does not capture the acidity under irradiation. The alternative measures of acidity, e.g., radiolytic acidity $\pi*$ and radiolytic ion product K_W* , were recommended since they account for radiation-induced alterations of both $H+$ and $OH−$ concentration. They further demonstrated that adding pH-irrelevant solutes such as LiCl, LiBr, or $LiNO_3$ can trigger a significant change in $\pi*$, thus tailor the acidity. Overall, the work was done carefully and systematically. I recommend publication in The Journal of Physical Chemistry Letters after the author address the minor concerns as it follows.

In practical experiments, H_2 gases are often generated. And, O_2 gases can also form from the electron beam irradiation of water. How does the gas formation (including formation kinetics) impact the $H+$ and $OH−$ concentration?

Reviewer: 2

Comments to the Author
Review of ” Tailoring the Acidity of Liquid Media with Ionizing Radiation: Rethinking the Acid-Base Correlation Beyond pH” by Fritsch et al.

The paper thoroughly discusses and make a simulation study of the concentration of $H+$ and $OH-$ during radiolysis of water, finding that pH is not a well defined measure of the acidity/alkalinity of a solution when irradiated strongly.

Strangely this has not been noticed or discussed in published literature to my knowledge, while pH variation upon radiation is discussed in several works. Hence it is appropriate and timely to clarify this aspect as done here. Covering both electron and x-rays this paper will find a wider audience.

I find the paper well written, with minor questions and improvements noted below to address before publication:

1) Other things than irradiation of water can make variations in the dissociation constant e.g.:

a. Wien effect https://www.nature.com/articles/s41467-022-33451-1 and https://www.princeton.edu/~fhs/fhspapers/fhspaper93.pdf

b. Temperature dependence J. Phys. Chem. Ref. Data, Vol. 35, No. 1, 2006

c. Electrolysis https://iopscience.iop.org/article/10.1149/2.0312001JES

d. Likely there are many more examples.

I find it would strengthen the paper to provide a perspective of how the dissociation can be altered in more general terms.

Include in this pH is handled in these situations, to see if your concept of pi* is new or other measures are used or maybe suitable (I think your Pi is fine, but if there already is a standard term or measure typically used then that would be worth using here too or at least mention).

2) I think it would be appropriate to briefly discuss on the scale of possible variations in Kw, how big are the changes you observe? E.g. is a given dose rate a perturbation much larger than say heating to 100C – simply to provide a sense of how strong this effect is compared to other such effects.

3) You state that “Furthermore, H+ and OH- themselves are primarily generated” but it is not clear what is meant by this – refer to SI section on this.

4) G values may however change with the liquid conditions – has that been checked? If the G-value change with pH or Pi, then the conclusions will not hold. An example can be found in e.g. https://link.springer.com/article/10.1163/156856700X00525 and https://cdnsciencepub.com/doi/pdf/10.1139/v64-389 . This is an important assumption that needs to be commented and checked if it holds.

5) In the abstract consider changing “pH irrelevant” to “pH neutral”?

6) Equation (2) should follow from your rates 1 and 2 in table s2 – please make the connection explicit. Then it also becomes more understandable that adding new reactions and rates changes the apparent Kw rather than say changes in a(H2O)

7) Make SI sections numbered and refer to the sections number in the main text to ensure the reader understands what you are referring to.

8) Fig 2b add legend for both K* and Kw nonirradiated

9) Fig 3b maybe add a horizontal line at pH 7 and write this is shown in Fig 4

10) You state electrode potentials for Li+ and strongest reductant, but since these are close in potentials compared to 25mV thermal energy and the concentrations are not standard, please make an explicit calculation of the expected chemical equilibrium concentrations to assess if this should be neglected or included, as your many coupled reactions likely contain reactions that are less relevant than this one.

11) Apart from the chemical reactions with Li+, radiolysis could cause direct changes – are there any relevant Li+ processes with G-values?

12) P10 line 13 you state reaction rate constants, but HBr has different value in SI table S2

Author's Response to Peer Review Comments:

Reviewer(s)' Comments to Author:

Reviewer: 1

Comments:
In this paper, Fritsch et al reported their study of ionizing radiation induced acidity of liquids. This is a topic that has not been well understood… Nowadays, liquid phase electron microscopy and in-situ X-ray characterization methods have attracted significant interest. An understanding of the radiation induced changes in chemistry, or more specially acidity variation is of great interest. In this paper, the authors used kinetic modeling of radiation chemistry and showed that that pH does not capture the acidity under irradiation. The alternative measures of acidity, e.g., radiolytic acidity π∗ and radiolytic ion product KW* , were recommended since they account for radiation-induced alterations of both H+ and OH− concentration. They further demonstrated that adding pH-irrelevant solutes such as LiCl, LiBr, or LiNO3 can trigger a significant change in π∗, thus tailor the acidity. Overall, the work was done carefully and systematically. I recommend publication in The Journal of Physical Chemistry Letters after the author address the minor concerns as it follows.

In practical experiments, H2 gases are often generated. And, O2 gases can also form from the electron beam irradiation of water. How does the gas formation (including formation kinetics) impact the H+ and OH− concentration?

> We thank the reviewer for this comment. Gas dissipation into a bubble could indeed alter π^* and K_w^*. Ambrožič *et al.* (ref. 51 in the main manuscript) suggested a change of $c(H^+)$ and $c(OH^-)$ with increased pressure of an already present, bulk-like gaseous phase in contact with the liquid during LP-TEM. Yet, bubble formation itself is even more complicated due to the required supersaturation and the large Laplace pressure of nanobubbles.
> In our manuscript we focus on the impact of acidity under irradiation on homogeneous solutions without regarding heterogeneous processes such as precipitation of bubbles or nanocrystals. Nonetheless, we agree with the referee that bubble formation is of large interest for experimental discussions. Therefore, we **added the following statement** to page 12f of the main manuscript.
>
>> Moreover, radiation-induced formation of additional phases such as gas bubbles or nanocrystals could further alter the steady state concentrations of H^+ and OH^- and hence affect the acidity of the specimen solution.

Reviewer: 2

The paper thoroughly discusses and make a simulation study of the concentration of H+ and OH- during radiolysis of water, finding that pH is not a well defined measure of the acidity/alkalinity of a solution when irradiated strongly.

Strangely this has not been noticed or discussed in published literature to my knowledge, while pH variation upon radiation is discussed in several works. Hence it is appropriate and timely to clarify this aspect as done here. Covering both electron and x-rays this paper will find a wider audience.

I find the paper well written, with minor questions and improvements noted below to address before publication:

1) Other things than irradiation of water can make variations in the dissociation constant e.g.:
a. Wien effect https://www.nature.com/articles/s41467-022-33451-1 and https://www.princeton.edu/~fhs/fhspapers/fhspaper93.pdf
b. Temperature dependence J. Phys. Chem. Ref. Data, Vol. 35, No. 1, 2006
c. Electrolysis https://iopscience.iop.org/article/10.1149/2.0312001JES
d. Likely there are many more examples.

I find it would strengthen the paper to provide a perspective of how the dissociation can be altered in more general terms.
Include in this pH is handled in these situations, to see if your concept of pi* is new or other measures are used or maybe suitable (I think your Pi is fine, but if there already is a standard term or measure typically used then that would be worth using here too or at least mention).

We thank the reviewer a lot for this question, as it shows the general need to characterize activity beyond pH. Thus, we would like to address the mentioned effects subsequently.

a) Wien effect:

Indeed, the mentioned work shows that for weakly bonded electrolytes, electrostatic fields enhance the rate of ionization and consequently the electrolytes ionization constant increases. This was recently investigated and quantified for water at different pH values by Cai *et al.* (https://www.nature.com/articles/s41467-022-33451-1). However, the authors do not provide a constructive definition of an alternative measure of acidity. We believe that this is due to the fact that here the water splitting is producing identical amounts of H^+ and OH^- ions -which is not necessarily the case for radiation chemistry as we show in our manuscript.

b) Bandura and Lvov (J. Phys. Chem. Ref. Data, Vol. 35, No. 1, 2006) show theoretical calculations of K_W as a function of temperature. Again, no redefinition of pH is strictly required as the processes defining the acidity do not bypass autoprotolysis but change its equilibrium constant. However, these thermally induced changes are notable, yielding ion products that are comparable to radiation chemistry values up to about 10^5 Gy/s. We consider this information to be highly beneficial for our manuscript. Therefore, we included them in Figure 2(b). It now displays as:

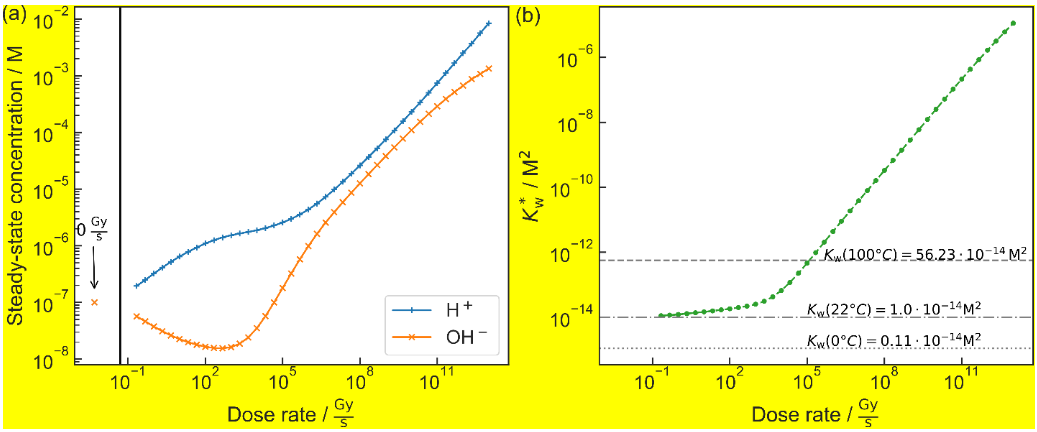

Figure 1: (a) Steady-state concentrations of H^+ and OH^- in pure, aerated water and (b) respective radiolytic ion product, both as a function of the dose rate of electron irradiation compared to changes in K_W with temperature.

Likewise, a statement was added to page 7 of the main manuscript:

> For dose rates up to about 100 kGy·s^{-1} thermally induced changes in water at ambient pressure could cause similar deviations (Figure 2(b)). However, due to the formation multiple primary species (eq. (5)), K_W^* is the consequence of diverging values for $c(H^+)$ and $c(OH^-)$. This is fundamentally different in the case of thermally excited ion products as here charge and mass balance demand that $c(H^+)$ and $c(OH^-)$ change equally[35].

Moreover, the work of Bandura and Lvov (J. Phys. Chem. Ref. Data, Vol. 35, No. 1, 2006) was added as ref. 35.

c) As pointed out by the reviewer, water electrolysis can also affect the acidity. As shown by Feng *et al.* (https://iopscience.iop.org/article/10.1149/2.0312001JES), pH / pOH could become a function of a distance from the respective electrodes. Again, no alternative measure of acidity is provided. However, as the underlying electrochemical principles can become non-trivial, we do not discuss this issue in depth within the present manuscript.

Again, we acknowledge the reviewer's suggestion to broaden the range of effects that need to be addressed when considering influences on the acidity of water. Therefore, we included a paragraph to our discussion section to address these points appropriately (p. 15):

> Besides radiolysis, other effects can influence K_W and consequently the acidity of water, such as temperature (see above) or the Wien effect[49]. Water electrolysis also exhibits the potential to locally alter the acidity towards a non-equilibrium state, especially dominant in proximity to

the electrode surface[50]. In such cases, regarding the logarithmic ratio $c(H^+)$ and $c(OH^-)$ could be potentially beneficial, as well.

The work of Cai *et al.* is referenced as ref 49 (https://www.nature.com/articles/s41467-022-33451-1) whereas ref 50. denotes the paper of Feng *et al.* (https://iopscience.iop.org/article/10.1149/2.0312001JES) that has been suggested by the reviewer.

2) I think it would be appropriate to briefly discuss on the scale of possible variations in Kw, how big are the changes you observe? E.g. is a given dose rate a perturbation much larger than say heating to 100C – simply to provide a sense of how strong this effect is compared to other such effects. And discuss what causes changes in the non-irradiated Kw vs your case, and if there are similarities and differences. Could there be more effects that would need to be considered learning from the temperature induced Kw changes or others?

We agree with the reviewer that such a comparison is increasing the quality of our manuscript. Thus we added the above-mentioned paragraph on temperature-related effects (p 7):

For dose rates up to about 100 $kGy \cdot s^{-1}$ thermally induced changes in water at ambient pressure could cause similar deviations (Figure 2(b)). However, due to the formation multiple primary species (eq. (5)), K_W^* is the consequence of diverging values for $c(H^+)$ and $c(OH^-)$. This is fundamentally different in the case of thermally excited ion products as here charge and mass balance demand that $c(H^+)$ and $c(OH^-)$ change equally[35].

3) You state that "Furthermore, H+ and OH- themselves are primarily generated" but it is not clear what is meant by this – refer to SI section on this.

We included the suggested reference to our supplementary information into the main manuscript. The statement now reads:

Furthermore, H^+ and OH^- themselves are primarily generated (eq. (5), see supporting information section S1.3) […].

4) G values may however change with the liquid conditions – has that been checked? If the G-value change with pH or Pi, then the conclusions will not hold. An example can be found in e.g. https://link.springer.com/article/10.1163/156856700X00525 and https://cdnsciencepub.com/doi/pdf/10.1139/v64-389 . This is an important assumption that needs to be commented and checked if it holds.

We agree that any alterations from pristine water can alter the G-values. This is why we limited our simulation to parameters where these alterations are minor. Moreover, we additionally labelled any data point coming close to this limit within our manuscript with hollow markers. This is written within the computational methods section:

Here, we assume sole interaction of radiation with water, for which *G*-values are well-known (Table S1 of the supporting information). […] Simulation results where the amount of radiolytic products in steady state exceeds 1% of the water concentration are labelled appropriately, as here, the assumption of radiation only interacting with the solvent becomes questionable.

To make this clearer, we altered the related paragraph in the discussion on page 13. It now reads:

Mind also that any change of the solvent limits the validity of the assumption that the radiation only interacts with water. This is particularly relevant for high dose rates (usually above ~10^{13} $Gy \cdot s^{-1}$)[14] where solvent consumption becomes notable.

5) In the abstract consider changing "pH irrelevant" to "pH neutral"?

We changed the wording accordingly. The statement now reads:

Moreover, we demonstrate that adding pH-neutral solutes such as LiCl, LiBr, or $LiNO_3$ can trigger a significant change in π^*.

6) Equation (2) should follow from your rates 1 and 2 in table s2 – please make the connection explicit. Then it also becomes more understandable that adding new reactions and rates changes the apparent Kw rather than say changes in a(H2O)

We thank the reviewer for this suggestion. As the formulation of the equilibrium constant K can equivalently derived from the rate constants, we reformulated equation (2) and its introductory paragraph as suggested:

Considering the law of mass action, an equilibrium constant K can be formulated. This can be achieved by regarding the ratio of forwards and backwards reaction k_f/k_b, as well as via the activity α of the respective species:

$$K = \frac{k_\mathrm{f}}{k_\mathrm{b}} = \frac{\alpha(\mathrm{H}^+)\cdot\alpha(\mathrm{OH}^-)}{\alpha(\mathrm{H_2O})} \quad (2)$$

7) Make SI sections numbered and refer to the sections number in the main text to ensure the reader understands what you are referring to.

We labelled the SI sections as suggested and referenced them appropriately in the main manuscript.

8) Fig 2b add legend for both K* and Kw nonirradiated

Thank you for your support in figure design within our manuscript. As we redesigned Figure 2(b) with regards to your above-mentioned comment, we consider it now to be well understandable:

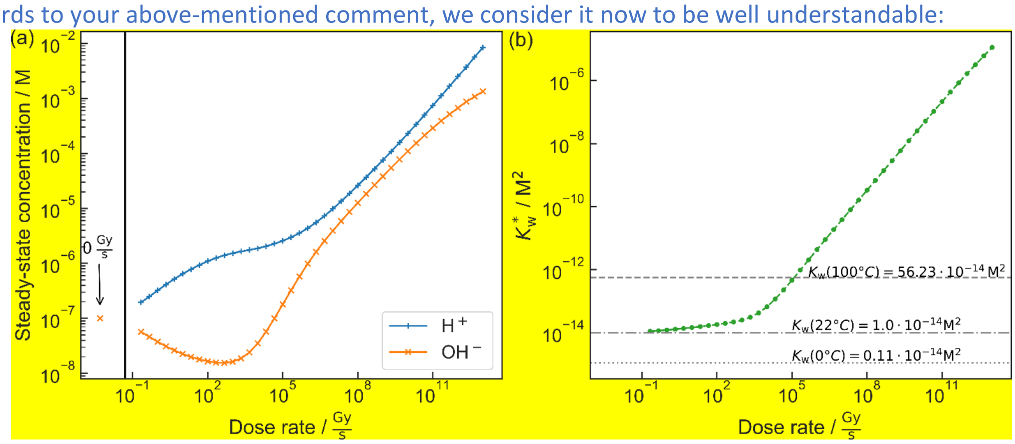

Figure 2: (a) Steady-state concentrations of H^+ and OH^- in pure, aerated water and (b) respective radiolytic ion product, both as a function of the dose rate of electron irradiation compared to changes in K_W with temperature.

9) Fig 3b maybe add a horizontal line at pH 7 and write this is shown in Fig 4

Thank you for the suggestion. However, as we already included level-lines, we consider this graphic to be very comprehensive already. Instead, we added a note in the Figure caption:

A crosscut at pH 7 can be found in Figure 4.

10) You state electrode potentials for Li+ and strongest reductant, but since these are close in potentials compared to 25mV thermal energy and the concentrations are not standard, please make an explicit calculation of the expected chemical equilibrium concentrations to assess if this should be neglected or included, as your many coupled reactions likely contain reactions that are less relevant than this one.

We agree that in general, radiation chemistry is not at standard conditions. However, to our knowledge this does not affect thermodynamics of species which are not directly ionized. As written above, all simulations are performed well within the dilute solution approximation so that the raised

objections are minor. Nonetheless, the stated potentials are indeed close. To access a possible reduction of Li^+ we performed simulations for Na^+ which exhibits a lower standard potential than Li^+ and is known to be reducible by solvated electrons under elevated OH^- concentrations (Telser and Schindewolf, 1986, https://doi.org/10.1021/j100412a097).

To estimate the maximum error that could be introduced in our simulation, we performed additional simulations for 10 mM additives of Na^+ (to illustrate the alteration introduced purely by the alkali ion), NaCl, NaBr, and $NaNO_3$. 10 mM is the highest concentration used in the simulations of the main manuscript. These simulations were performed for the electron-irradiated case.

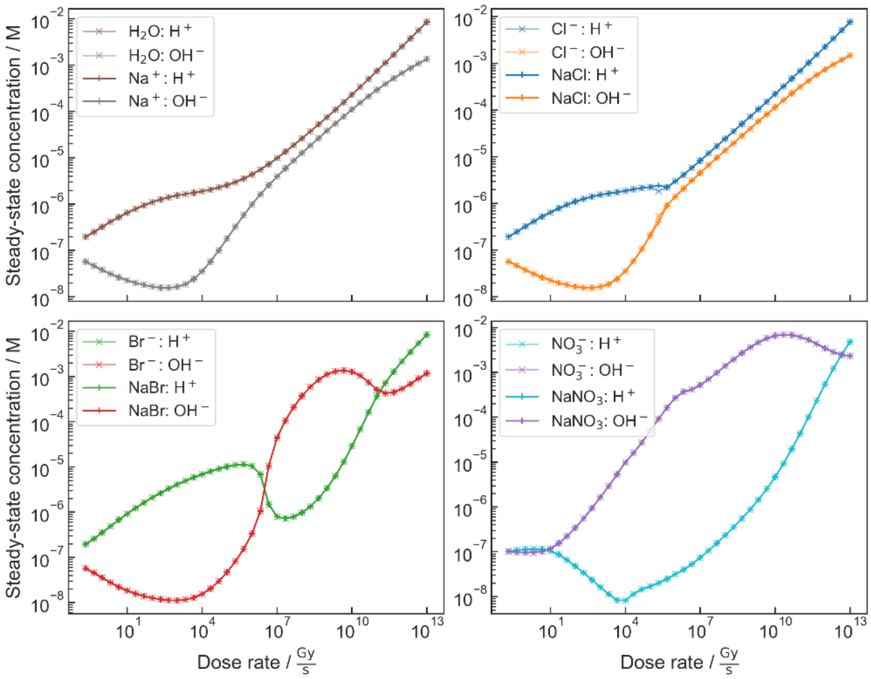

Evidently, the changes in the evolution of H^+ and OH^- concentrations by introducing Na^+ are negligible within the whole dose rate range. Consequently, the impact of Li^+ is even less relevant for the acidity under irradiation.

To account for the concern raised by the reviewer, this consideration is now included in the supplementary information:

IMPACT OF ALKALI METALS ON THE ACIDITY UNDER IRRADIATION

Albeit the standard potentials of Li^+ is slightly higher than the reductive potential of solvated electrons (see main manuscript), the difference is close to thermal energy. To demonstrate that its impact is negligible for the discussion within this work, we simulated an extreme case scenario, in which we assume the reactivity of Li^+ to be similar to the one of Na^+. For the latter,

Tesler and Schindewolf[5] measured a reduction by solvated electrons. They reported the reaction:

$Na^+ + e_h^- \rightarrow Na$ with a rate constant of $2 \cdot 10^4\ (Ms)^{-1}$

As decay, the reaction with H_2O was given within the same manuscript as:

$2\ Na + 2\ H_2O \rightarrow H_2 + 2\ Na^+ + 2\ OH^-$ with a rate constant of $1.5 \cdot 10^9\ (Ms)^{-1}$

Particularly the latter reaction has the potential to alter the acidity under irradiation. However, to simulate elementary steps only, the latter reaction was considered in the form of

$Na + H_2O \rightarrow H + Na^+ + OH^-$ with a rate constant of $1.5 \cdot 10^9\ (Ms)^{-1}$, because the recombination of $2\ H \rightarrow H_2$ (Reaction 34 in Table S2) is more than five times faster than the value given here, so that the oxidation of Na was assumed to be rate-determining.

By incorporating these proposed reactions (Table S6) we simulate the evolution of H^+ and OH^- steady state concentrations of 10 mM solutions of pure Na^+, NaBr, NaCl, and $NaNO_3$ under 300 keV electron irradiation (Figure S9). It is evident that Na^+ does not alter the obtained concentrations when considered as a hypothetical stand-alone reactant to pure water. This does not change notably when more realistic scenarios (NaBr, NaCl, $NaNO_3$) are regarded. Consequently, a change of Li^+ is likely to be negligible throughout all simulations within this manuscript.

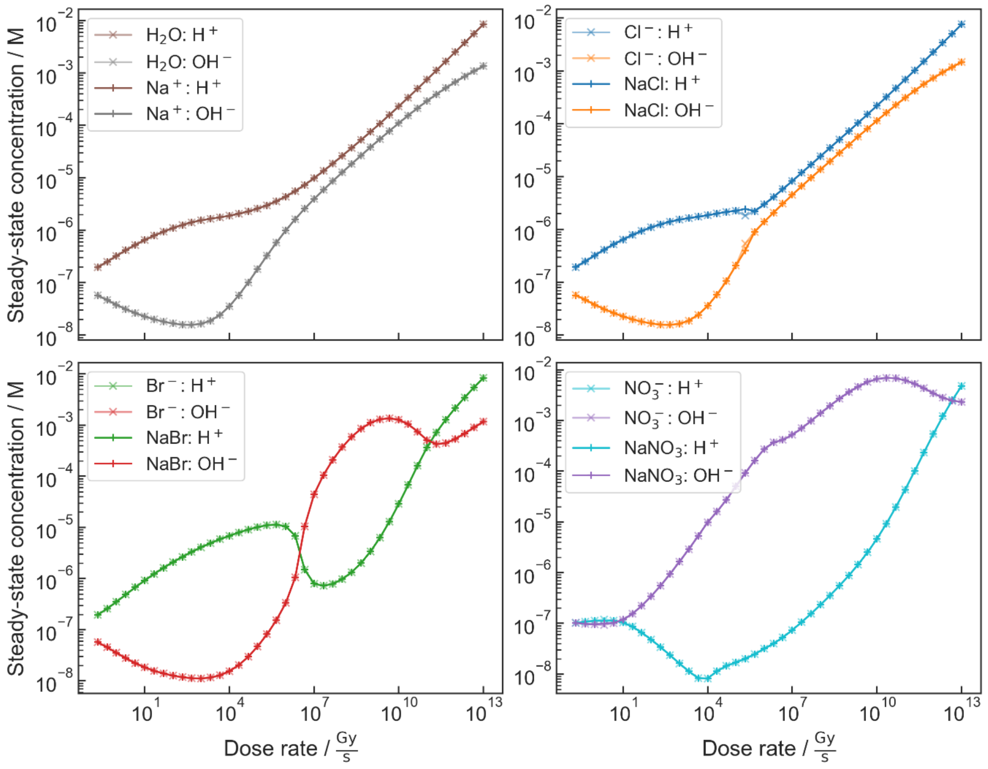

Figure S1: Steady-state evolutions of H^+ and OH^- of pure water, and 10 mM Cl^-, Br^-, or NO_3^- containing solutions with and without additional 10 mM Na^+ present.

Table S6: Kinetic model used to describe the radiolysis of Na^+-containing aqueous solutions. Here, k denotes the respective kinetic constant in units of $mol^{-n+1}\ L^{3(n-1)}\ s^{-1}$, where n denotes the reaction order. Please refer to Table S2 for the first 83 reactions.

	Reaction			k	Source
84	$Na^+ + e_h^-$	→	Na	$2 \cdot 10^4$	[5]
85	$Na + H_2O$	→	$H + Na^+ + OH^-$	$1.5 \cdot 10^9$	Assumed as rate-determining elementary step, after ref.[5]

Furthermore, a statement was added to the main manuscript:

> Furthermore, as demonstrated, even additives considered as non-reactive, can drastically change the steady-state chemistry at hand. Thus, any extrapolation should be performed cautiously. In this context, it is advised to double-check the assumption of negligible Li^+ influence on the acidity under irradiation. To do so, we considered a reactivity similarly to the slightly more noble Na^+ (see Figure S9 and Table S6 in the supplementary information)[40]. Evidently, the impact on $c(H^+)$ and $c(OH^-)$ remains negligible. As Tesler and Schindelwolf[40] measured similar rates for Cs^+, as well, we assume that our findings would also hold for CsCl, CsBr, and $CsNO_3$-containing solutions.

The added reference in in both documents correspond to the aforementioned work of Telser and Schindewolf (https://doi.org/10.1021/j100412a097).

11) Apart from the chemical reactions with Li+, radiolysis could cause direct changes – are there any relevant Li+ processes with G-values?

We agree with the reviewer that Li^+ can alter the formation of initial and primary species. However, as written above, this can be neglected for highly diluted solutions. This is the case for all simulations performed in the present manuscript.

12) P10 line 13 you state reaction rate constants, but HBr has different value in SI table S2

We thank the reviewer for such detailed and comprehensive evaluation of our work. Thus, we double-checked the stated rate constants for deprotonation of HCl, HBr, and HNO_3, in both, the main manuscript and the supplementary information and can confirm that they are identical in all cases.
